# Integrating Artificial Intelligence in Bronchoscopy and Endobronchial Ultrasound (EBUS) for Lung Cancer Diagnosis and Staging: A Comprehensive Review

**DOI:** 10.3390/cancers17172835

**Published:** 2025-08-29

**Authors:** Sebastian Winiarski, Marcin Radziszewski, Maciej Wiśniewski, Jakub Cisek, Dariusz Wąsowski, Dariusz Plewczyński, Katarzyna Górska, Piotr Korczyński

**Affiliations:** 1 Department of Thoracic Surgery, National Medical Institute of the Ministry of the Interior and Administration, 02-507 Warsaw, Poland; marcin.radziszewski@pimmswia.gov.pl (M.R.); dariusz.wasowski@pimmswia.gov.pl (D.W.); 2 Department of Histology and Embryology, Medical University of Warsaw, 02-004 Warsaw, Poland; 3 Faculty of Mathematics and Information Science, Warsaw University of Technology, 00-662 Warsaw, Poland; m.wisniewski@datascience.edu.pl (M.W.); d.plewczynski@cent.uw.edu.pl (D.P.); 4 Faculty of Computer Science, Polish-Japanese Academy of Information Technology, 02-008 Warsaw, Poland; cisekjakub@wp.pl; 5 Department of Pulmonary Diseases, Thoracic Oncology and Transplantology, National Medical Institute of the Ministry of the Interior and Administration, 02-507 Warsaw, Poland; katarzyna.gorska2@pimmswia.gov.pl (K.G.); piotr.korczynski@pimmswia.gov.pl (P.K.)

**Keywords:** artificial intelligence (AI), deep learning (DL), endobronchial ultrasound (EBUS), endobronchial ultrasound-guided transbronchial needle aspiration (EBUS-TBNA), bronchoscopy, lung cancer

## Abstract

Artificial intelligence (AI) is increasingly investigated as a potential adjunct in the diagnosis and staging of lung cancer, particularly through integration with bronchoscopy and endobronchial ultrasound (EBUS). Deep learning models have been applied to modalities such as white-light imaging, autofluorescence bronchoscopy, and spectroscopy, with the aim of assisting lesion detection, standardizing interpretation, and reducing interobserver variability. AI has also been explored in EBUS for lymph node assessment and guidance of transbronchial needle aspiration (EBUS-TBNA), with preliminary studies suggesting possible improvements in diagnostic yield. However, current evidence remains largely confined to small, retrospective, single-center datasets, often reporting performance under idealized conditions. External validation is rare, reproducibility is undermined by a lack of data and code availability, and workflow integration into real-world bronchoscopy practice has not been demonstrated. As such, most systems should still be regarded as experimental. Translating AI into routine thoracic oncology will require large-scale, prospective, multicenter validation studies, greater data transparency, and careful evaluation of cost-effectiveness, regulatory approval, and clinical utility.

## Simple Summary

Interobserver variability remains a significant challenge in the accurate diagnosis and staging of lung cancer. This review analyzes existing artificial intelligence (AI) models for bronchoscopy and endobronchial ultrasound (EBUS) visual analysis, bridging engineering innovations with clinical needs to enhance diagnostic precision, standardize evaluations, and optimize procedural guidance. By providing a structured overview of current approaches, it lays the foundation for future system development and highlights the translational potential of these technologies to advance routine practice in thoracic oncology.

## 1. Introduction

Bronchoscopy, including endobronchial ultrasound (EBUS) and emerging robotic-assisted techniques, plays a central role in the diagnosis, staging, and management of lung cancer and mediastinal lymphadenopathy [1]. These minimally invasive procedures offer real-time imaging, precise tissue sampling, and guided navigation, all of which are critical for early diagnosis and informed clinical decision-making. Their effectiveness in evaluating central airway lesions and mediastinal lymph nodes has made them indispensable tools in thoracic oncology [2]. However, challenges such as operator-dependent variability, procedural complexity, and diagnostic uncertainty due to image interpretation and navigation issues persist [3,4].

Recent advances in artificial intelligence (AI) and machine learning (ML) are ushering in new possibilities for medical imaging and interventional pulmonology [5]. Specifically, deep learning (DL) and convolutional neural networks (CNNs) have demonstrated considerable promise in tasks such as automated image analysis, lesion detection, and clinical decision support. These AI technologies hold significant potential to enhance procedural accuracy and reproducibility, thereby reducing inter-operator variability. In the context of bronchoscopy and EBUS, AI can assist with lesion localization, biopsy tool guidance, and real-time interpretation of ultrasound images [6].

The integration of AI into bronchoscopy workflows could help address longstanding challenges in the field. By enabling automated image recognition, trajectory optimization, and real-time feedback, AI-enhanced systems could improve biopsy yield, shorten procedural times, and increase diagnostic confidence [7]. Additionally, AI may aid in standardizing procedures across different institutions and operator experience levels, thereby enhancing training, improving procedural safety, and ensuring consistent care. Notably, AI also holds promise in the analysis of cytological and histological specimens, further supporting diagnostic accuracy and overall procedural efficiency [8].

Despite promising advances, the clinical adoption of AI in bronchoscopy and EBUS remains limited. The available evidence is heterogeneous, and large-scale validation studies are still relatively scarce. This review provides a comprehensive overview of AI applications in bronchoscopy and EBUS, with a particular emphasis on lung cancer diagnosis and staging. It summarizes recent technological innovations, evaluates the current body of clinical evidence, and highlights future research directions, underscoring the translational potential of integrating engineering advances into clinical practice. The review is informed by a systematic literature search conducted in accordance with PRISMA guidelines, in which 2116 articles were screened and 35 studies were ultimately included. All included studies were assessed for risk of bias using the QUADAS-2 tool. To enhance readability, the findings are presented in a narrative format, structured around the results of the systematic review, with full methodological details provided in the Supplementary Material.

## 2. Core Concepts of Artificial Intelligence

**Figure 1:**
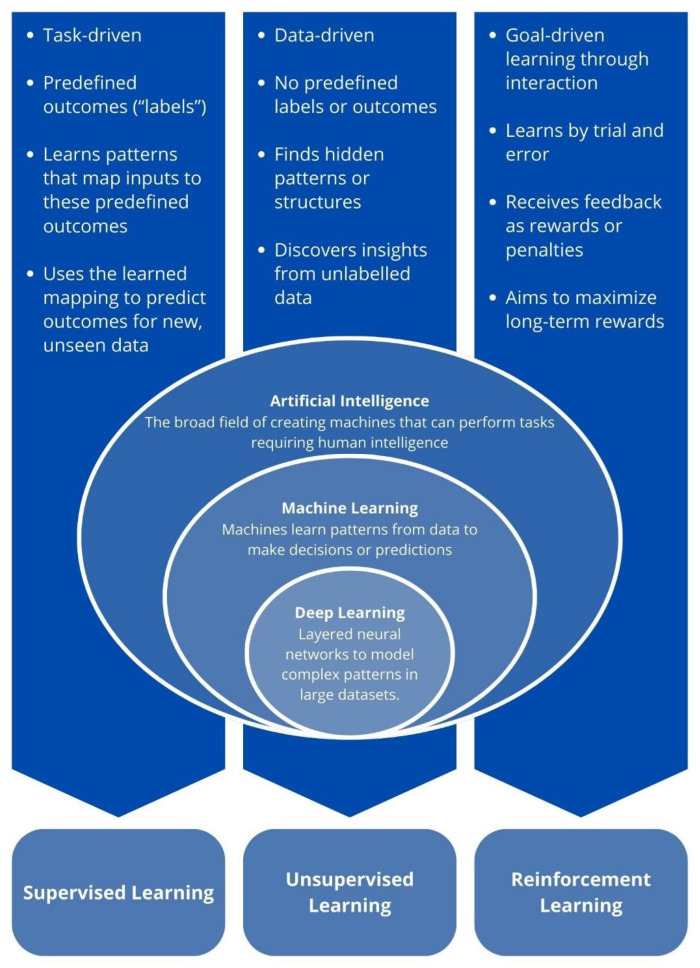
Hierarchical relationship between AI, ML, and DL, highlighting the three primary learning paradigms: supervised, unsupervised, and reinforcement learning. DL has gained particular prominence in medical imaging for its ability to improve accuracy, automate image analysis, and enhance diagnostic capabilities. Created with Canva.com.

AI is a rapidly evolving field focused on developing systems capable of performing tasks that traditionally require human intelligence, such as reasoning, decision-making, pattern recognition, and learning [9]. Within AI, ML emphasizes algorithms that enable systems to learn from data and adapt performance without explicit task-specific programming [10]. Unlike conventional software, which relies on predefined rules, ML models dynamically adjust their behavior based on input data. Figure 1 illustrates the fundamental concepts in AI, highlighting the principal learning approaches.

ML approaches are commonly classified into three main categories: supervised learning, unsupervised learning, and reinforcement learning [11]. In supervised learning, models are trained on labeled datasets, where both inputs and corresponding outputs are known, allowing the algorithm to learn mappings between them [12]. This method is particularly useful for tasks such as image classification, outcome prediction, and diagnostics. In contrast, unsupervised learning analyzes unlabeled data to uncover hidden patterns, groupings, or anomalies, supporting applications such as identifying novel disease phenotypes, stratifying patient populations, and discovering subtypes [13]. Reinforcement learning, the third paradigm, involves models that learn through trial and error in dynamic environments, guided by rewards or penalties. This strategy is valuable for sequential decision-making, including optimizing treatment strategies over time and improving robotic performance in surgery [14].

DL, a specialized subset of ML, employs multilayered artificial neural networks (ANNs) to progressively extract higher-level features from raw data [15]. Unlike traditional ML, which often depends on manual feature engineering, DL models autonomously learn and refine features during training, making them particularly effective for unstructured data such as medical images, natural language, and audio signals [11]. Inspired by the human brain, DL architectures are composed of interconnected nodes arranged in input, hidden, and output layers [16]. Each layer transforms data into increasingly abstract representations: in medical imaging, for example, early layers detect edges, while deeper layers capture textures or lesion morphology. This hierarchical learning enables DL models to excel in disease classification, lesion detection, and image segmentation across radiological, pathological, and endoscopic domains [17].

Among DL methods, CNNs are especially powerful for visual data analysis [18]. Their convolutional layers scan images to detect spatial and hierarchical features, enabling direct interpretation of imaging modalities such as computed tomography (CT), ultrasound, and X-ray [19].

### Critical Overview of Artificial Intelligence Applications in Medicine

While AI holds great promise in healthcare, its implementation requires critical evaluation. In medical imaging and diagnostics, algorithms show strong performance in disease detection and classification, yet challenges of data bias, transparency, and reproducibility limit clinical adoption [19]. In research and drug discovery, AI accelerates target identification and trial design but demands rigorous validation to ensure generalizability [20]. Invasive procedures and robotic platforms benefit from enhanced precision and reduced risk, though barriers such as cost, workflow integration, and surgeon training remain [21].

For patient care and rehabilitation, AI enables personalization and monitoring but raises ethical concerns regarding privacy, autonomy, and unequal access [22]. Administrative applications improve efficiency but introduce cybersecurity risks and potential workforce displacement [23]. Thus, despite its transformative potential, AI integration in healthcare must be accompanied by careful consideration of its limitations, risks, and ethical implications.

**Figure 2:**
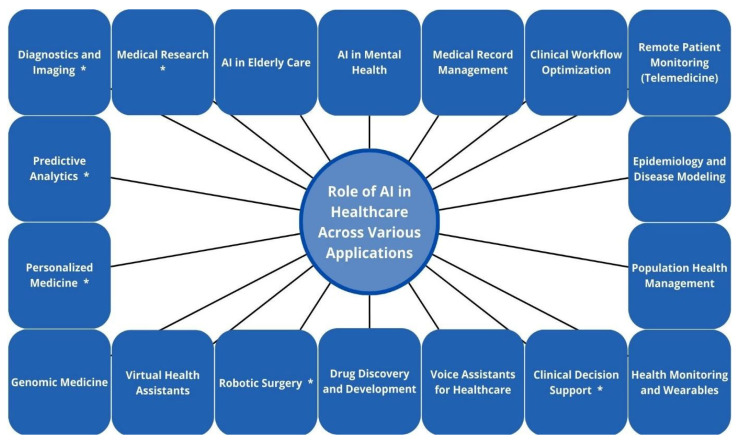
Diverse applications of AI in healthcare, with select domains marked by an asterisk (*) to indicate relevance to bronchoscopy and EBUS. Among these, diagnostics and imaging are most directly applicable, where AI supports lesion detection, segmentation, and classification. While these tools show promise, their effectiveness depends on access to robust datasets and rigorous clinical validation. Clinical decision support can aid in biopsy target selection and malignancy risk assessment, though adoption is limited by workflow integration and clinician trust. In robotic surgery, AI enhances precision in bronchoscopic procedures but remains resource-intensive. Predictive analytics enables outcome forecasting, yet concerns about model generalizability persist. Other domains, such as medical research and personalized medicine, may benefit indirectly from bronchoscopy/EBUS data but require further translational development. Created with Canva.com.

In this context, the application of AI in thoracic oncology, particularly in lung cancer diagnosis, treatment, and monitoring, exemplifies both the extraordinary potential and the complex challenges inherent in the adoption of AI-driven technologies in clinical practice [24]. Notably, the incorporation of AI into bronchoscopy and EBUS has shown potential to enhance lesion detection, navigation, and lymph node assessment; however, its clinical utility remains contingent upon rigorous validation, standardization of protocols, and careful integration into existing procedural workflows [25]. Figure 2 presents the broad applications of AI in healthcare, with specific areas marked by an asterisk (*) to highlight their relevance to bronchoscopy and EBUS.

## 3. Artificial Intelligence in Bronchoscopy for Lung Cancer Diagnosis

Bronchoscopy remains a cornerstone in the diagnosis of endobronchial lesions, providing direct visualization and vision-guided tissue sampling. Despite its established clinical utility, diagnostic yield is influenced by tumor location, accessibility, and operator expertise, and is subject to interobserver variability. Conventional approaches, including bronchial washing, brushing, and endobronchial biopsy, demonstrate variable yields ranging from 48% to 74%. When used in combination, these techniques can increase the overall diagnostic yield to approximately 88% [26]. Findings from the AEGIS trials further underscore these limitations: among 639 patients, 43% of bronchoscopic examinations were non-diagnostic for lung cancer, and 35% of patients with benign lesions underwent additional invasive procedures following bronchoscopy [27].

Recent advances in AI may help address some of these challenges by enhancing diagnostic precision, reducing interobserver variability, and promoting more standardized decision-making [28]. Table 1 summarizes key studies that have developed AI-based models for bronchoscopy image analysis across multiple modalities, including white light bronchoscopy (WLB), autofluorescence bronchoscopy (AFB), narrow-band imaging (NBI), and Raman spectroscopy (RS), with a focus on their application in the detection and diagnosis of lung cancer.

To date, the highest reported accuracy in lesion detection using WLB has been 97.8%, achieved with the multiscale attention residual network (MARN), a CNN-based model developed by Sun et al. [29]. This model was trained on 2900 frames from 615 patients, with the raw data made publicly available; however, no external validation was performed. Notably, its accuracy decreased by approximately 5% when the model was applied to distinguish malignant from benign lesions.

In another study, Deng et al. applied a ResNet101-based model to WLB images, achieving an accuracy of 95.1% for lesion detection and further demonstrating that DL can also support pathological subtype classification of lung cancer [30]. In a cohort comprising 312 squamous cell carcinoma (SCC), 178 adenocarcinoma (AC), and 129 small cell lung cancer (SCLC) cases, their model achieved an overall accuracy of 60% for three-class classification, which improved to 74.5% when restricted to SCC versus AC. Importantly, the system outperformed junior physicians and achieved diagnostic performance comparable to that of senior clinicians in distinguishing malignant lesions. In parallel, Tan et al. developed a DenseNet-based CNN with transfer learning through sequential fine-tuning (SFT), which successfully differentiated cancerous from tuberculous lesions in WLB [31].

Recent efforts have explored advanced knowledge distillation frameworks to enhance lesion detection in WLB. Yan et al. proposed the Prior Knowledge Distillation Network (PKDN), which integrates color and edge priors with spatial and channel attention mechanisms to better focus on lesion regions. Trained on more than 2000 bronchoscopic images from 200 patients, PKDN achieved an accuracy of 94.8% and an AUC of 98.2%, outperforming several state-of-the-art baselines [32]. Similarly, Liu et al. introduced the knowledge distillation–based memory feature unsupervised anomaly detection (KD-MFAD) model, which incorporates a downward deformable convolution (DDC) module and a convolutional block memory matrix (CB-Mem) to capture subtle airway abnormalities. On a self-built bronchoscopy dataset, KD-MFAD achieved an accuracy of 93.3% with an AUC of 97.6%, demonstrating robustness across both internal and external test sets [33]. Collectively, these studies indicate that knowledge distillation and anomaly detection strategies can achieve accuracies approaching 95% in bronchial lesion detection, while also improving model generalizability and interpretability.

A critical step in advancing AI-assisted bronchoscopy has been the introduction of publicly available datasets, which help overcome the limitations of restricted, institution-specific collections. The BI2K dataset, introduced alongside the MARN model, comprises 2900 bronchoscopic images from 615 patients and remains one of the largest open-access WLB resources to date, enabling reproducible benchmarking of lesion detection algorithms [29]. More recently, the BM-BronchoLC dataset has further enriched the field by providing meticulously annotated data from 208 patients (106 with lung cancer and 102 without), including detailed labels for both anatomical landmarks and airway lesions curated by senior bronchoscopists [34]. Together, these datasets provide diverse, high-quality material for training and validation, fostering transparency and comparability across studies. Their utility has already been demonstrated in the development of advanced detection models, such as BrYOLO-Mamba, which leverages optimized structured state-space modules to improve tracheal lesion detection, achieving significant performance gains while reducing computational cost [35].

In AFB, early studies confirmed the feasibility of computer-based lesion detection and classification. Chang et al. developed an automated video analysis pipeline using nearly 40,000 frames, achieving lesion detection accuracies exceeding 97%, although the dataset was limited to only four patients, thereby constraining generalizability [36]. Similarly, Haritou et al. proposed a texture- and color-based image analysis tool that achieved 95.4% accuracy in distinguishing malignant lesions from false positives caused by inflammation, though their evaluation was restricted to eleven patient cases [37]. Beyond detection, Feng et al. demonstrated that CAD systems applied to AFB images could also support pathological subtype classification, successfully differentiating AC from SCC with an accuracy of 83% (AUC of 81%) [38]. More recently, Chang et al. introduced ESFPNet, a transformer-based segmentation model trained on the first publicly available AFB dataset (20 patients), enabling real-time lesion detection and segmentation. ESFPNet achieved a Dice coefficient of 0.824 and an IoU of 0.707, outperforming established architectures such as UNet++ and CaraNet, while reducing computational cost [39].

**Table 1:** This table presents a curated summary of recent research studies leveraging AI for lung cancer diagnosis using bronchoscopy and related imaging modalities. Not all articles reported both the size of the patient cohort and the number of frames derived from the study population. It is important to note that a large dataset of frames generated from a small number of patients may lead to inflated accuracy estimates. Similarly, choosing only one frame per case may decrease the representativeness of the study. Owing to substantial heterogeneity in performance reporting, accuracy, sensitivity, and specificity were selected as the primary comparative metrics. For studies in which these parameters were not reported, precision was included as an alternative.

Input Data Type	Diagnostic Task	Model Type	Unit of Analysis	Dataset Size	Ground Truth	Performance Metrics	External Validation	Data Availability	Year	Authors [Ref.]
WLB	Lesion detection	CNN	Frames	2908	Expert consensus	Acc: 93.3%	Yes	Public	2025	Liu et al. [33]
WLB	Lesion detection	CNN	Patients/Frames	615/2900	Histopathology	Acc: 97.8%	No	Public	2024	Sun et al. [29]
WLB	Lesion detection	CNN	Frames	28,032	Expert consensus	Acc: 83.3%, Sen: 79.3%, Spe: 86.1%	No	On request	2024	Cao et al. [35]
WLB	Lesion classification	CNN	Patients/Frames	208/2921	Histopathology	Acc: 82–94%	No	Public	2023	Vu et al. [34]
WLB	Lesion detection	CNN	Patients/Frames	200/2029	Expert consensus	Acc: 94.8%	No	On request	2023	Yan et al. [32]
WLB	Lesion detection	CNN	Patients/Frames	818/2238	Histopathology	Acc: 95.1%, Sen: 97.8%, Spe: 83.3%	No	Not available	2022	Deng et al. [30]
WLB	Lesion classification	CNN	Patients	434	Histopathology	Acc: 82%	No	Not available	2018	Tan et al. [31]
AFB	Lesion detection	AE	Patients/Frames	20/685	Expert consensus	Prec: 86.2%	No	Public	2024	Chang et al. [39]
AFB	Lesion detection	SVM, ML	Patients/Frames	4/39,899	Expert consensus	Acc: ≥ 97%	No	Not available	2020	Chang et al. [36]
AFB	Lesion classification	ML	Patients	23	Histopathology	Acc: 83%, Sen: 73%, Spe: 92%	No	Not available	2018	Feng et al. [38]
AFB	Lesion classification	ML	Patients/Frames	11/715	Histopathology	Acc: 95.4%, Sen: 95.5%, Spe: 95.2%	No	Not available	2014	Haritou et al. [37]
NBI	Lesion detection	CNN	Patients/Frames	23/66,219	Expert consensus	Sen: 93%, Spe: 86%	No	Not available	2024	Daneshpajooh et al. [40]
RS	Lesion classification	ML	Patients/Spectra	70/78	Histopathology	Acc: 87.2%	No	On request	2024	Fousková et al. [41]

Abbreviations: convolutional neural network (CNN), autoEncoder (AE), support vector machine (SVM), machine learning (ML), white-light bronchoscopy (WLB), narrow-band imaging (NBI), autofluorescence bronchoscopy (AFB), Raman spectroscopy (RS), accuracy (Acc), sensitivity (Sen), specificity (Spe), precision (Prec).

AI models have also been extended to other bronchoscopic and optical modalities. In NBI bronchoscopy, Daneshpajooh et al. reported a two-stage DL-based system that achieved a sensitivity of 93% and specificity of 86% for lesion detection across 23 patient airway videos [40]. In parallel, RS has been investigated as a complementary diagnostic tool. A recent study demonstrated that an ML model applied to in vivo RS spectra attained a sensitivity of 89.7% and specificity of 84.6% for lung cancer diagnosis [41]. While NBI-based models primarily focus on localizing suspicious lesions, RS systems aim to characterize their molecular composition, highlighting their complementary potential in early lung cancer detection.

Overall, most AI models developed for bronchoscopy image analysis rely on DL, with a particular emphasis on CNN architectures. Both WLB- and AFB-based systems have shown strong potential for accurate lesion detection, including the recognition and classification of malignant lesions. Moreover, ML-based spectral classification of RS signals has demonstrated promise for malignancy detection without the need for biopsy. Nonetheless, the majority of models lack thorough clinical validation, which limits their integration into routine practice. Furthermore, although several publicly available datasets have recently been released, their number and diversity remain limited, continuing to restrict reproducibility and the standardized benchmarking of future models. In addition, the available studies carry considerable risk of bias, as they are most often single-center, retrospective, small-cohort investigations conducted under idealized conditions (Supplementary Figure S2). Prospective multicenter trials are essential to establish the validity and generalizability of these models before they can be implemented in clinical practice under regulatory approval.

### 3.1. The Role of Artificial Intelligence in Bronchonavigation

DL models are increasingly being explored as tools to enhance bronchoscopy, with potential applications in lesion detection, recognition, and navigation [42,43]. In principle, AI-assisted navigation in WLB could enable more precise lesion targeting and thereby improve the diagnostic yield of bronchoscopic biopsies. At present, however, these approaches remain largely experimental. For such systems to become clinically viable, AI models must first be trained to reliably identify a wide range of anatomical landmarks and bronchial branches throughout the airway tree [44,45].

**Figure 3:**
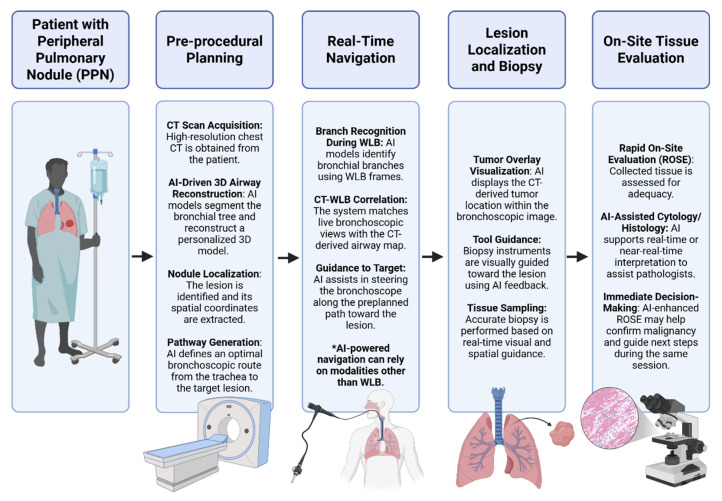
AI-enhanced workflow for WLB-based bronchonavigation in patients with peripheral pulmonary nodules (PPNs). The figure illustrates how AI can support each step of the procedure, from initial CT-based airway and lesion analysis to real-time guidance during bronchoscopy. AI models enable automated 3D reconstruction of the bronchial tree, accurate lesion localization, and path planning. During the procedure, WLB images are processed by AI to recognize anatomical landmarks, estimate bronchoscope position, and match the visual field to the preoperative CT map. This enables precise navigation to the target without reliance on expensive tracking systems or intraoperative radiation, underscoring the potential of AI to make advanced bronchoscopy more accurate, accessible, and efficient. Created with BioRender.com.

A commonly proposed framework begins with the generation of a patient-specific three-dimensional (3D) model of the bronchial tree, derived from CT data [46]. Within this framework, AI may support automated airway segmentation and 3D reconstruction [47,48]. In addition, accurate localization of the target lesion would need to be extracted from CT imaging to define an optimal navigation path. During the procedure, real-time navigation would depend on adherence to this predefined route. Several proof-of-concept AI models are under development to assist in recognizing bronchial branches in WLB frames. By aligning the observed bronchial anatomy with the CT-derived map, these systems could, in the future, enable progression through the airways and guide the bronchoscope along the planned path to the lesion, thereby facilitating accurate localization and precise tissue acquisition (Figure 3).

Even though this approach is still in its early phases, initial studies have yielded promising findings. Li et al. developed a CNN trained on 28,441 bronchial lumen images, achieving 91.8% accuracy in bronchial branch recognition under controlled conditions [49]. In clinical settings, however, performance declined to 82.7% for main and lobar bronchi and to 54.3% when segmental bronchi were included. Notably, clinical validation showed that three out of four physicians improved anatomical landmark recognition when assisted by the model. Similarly, Chen et al. designed a CNN that achieved 91% accuracy in identifying primary and secondary bronchial branches but encountered difficulty with segmental bronchi [50]. For comparison, physicians with more than six months of training achieved a mean accuracy of 84.33 ± 7.52%. In another study, Yoo et al. demonstrated that a CNN model outperformed most clinicians in identifying primary bronchi, performing comparably only to the most experienced physicians [51]. These findings underscore the promise of AI in bronchial lumen recognition, though accurate identification of all airway branches leading to a target lesion remains essential, and current AI models still fall short of expert human performance.

To overcome these challenges, ongoing research is exploring various models designed to estimate bronchoscope pose using WLB images alone [52,53]. A particularly promising direction involves neural radiance fields (NeRF), a DL technique capable of reconstructing highly detailed 3D scenes by mapping spatial coordinates and viewing angles to color and density from 2D images [54]. The long-term objective is to achieve cost-effective navigation without expensive tracking systems or intraoperative radiation-based imaging [55].

Beyond WLB-based navigation, AI is also being applied to other modalities. Early clinical trials have reported encouraging results for AI-powered fluoroscopy-guided bronchonavigation [56,57]. In addition, shape-sensing technologies, such as fiber Bragg grating (FBG) catheters combined with AI software, provide a radiation-free alternative with promising accuracy and procedural support [58]. These technologies are especially impactful when integrated into robotic bronchoscopy systems, which are increasingly regarded as platforms for future autonomous interventions [59].

Robotic technologies are rapidly transforming bronchoscopy by offering enhanced precision, reach, and consistency compared with manual techniques [60]. Initial robotic platforms improved access to peripheral airways but remained dependent on operator expertise [61]. More recent innovations have produced semi-autonomous systems that integrate AI-driven navigation with user control, enabling novice clinicians to safely access distal bronchi via shared-control algorithms and shape-sensing feedback [62]. Specialized robotic bronchoscopes are also being developed to address the unique challenges of mechanically ventilated patients in critical care settings [63]. At the forefront of innovation, fully autonomous systems are being explored using vision transformers (ViT), surgical workflow recognition, and self-supervised learning to support real-time airway segmentation, tool tracking, and clinical decision-making [64,65,66].

In summary, AI holds substantial promise for improving bronchonavigation by enhancing lesion localization, airway recognition, and procedural accuracy. Among available modalities, WLB-based navigation is particularly appealing due to its cost-effectiveness and accessibility. However, WLB-only approaches remain in the early stages of development and currently lack the precision, robustness, and hardware integration required for standalone clinical use. By contrast, AI-guided navigation systems leveraging fluoroscopy or shape-sensing technologies are more advanced and clinically reliable. Looking ahead, the integration of AI with robotic bronchoscopy platforms represents a transformative direction, with the potential to increase biopsy yield, improve accessibility, and ultimately enable autonomous bronchoscopic procedures.

### 3.2. Artificial Intelligence in Competency-Based Endoscopy Training

There is a growing shift in endoscopy education from traditional volume-based training toward competency-based approaches, with increasing emphasis on the acquisition of practical skills in controlled simulation environments (Table 2) [67,68,69]. AI has significant potential to accelerate this transformation by providing immediate, objective feedback to trainees and reducing reliance on specialists, whose availability for direct supervision is often limited [70].

A randomized controlled trial by Agbontaen et al. demonstrated that AI-guided bronchoscopy training enabled intensive care unit (ICU) professionals to perform simulated procedures more quickly and efficiently compared with those trained under expert supervision alone [28]. Early trials further suggest that AI-based training systems can help novice endoscopists inspect more airway segments in a systematic manner and improve procedural speed [71,72]. For example, a randomized controlled trial by Cold et al. involving 24 novice bronchoscopists found that the group trained with an ML model completed the final assessment significantly faster than the control group trained without AI support [73].

AI-guided training can also be delivered using virtual, CT-derived bronchial tree models, making simulation-based education more accessible, particularly in resource-limited settings [74,75]. Preliminary evidence indicates that AI not only accelerates the learning curve but also serves as a valuable tool for performance assessment during bronchoscopy training [76]. Integration with standardized assessment instruments, such as the bronchoscopy-radiologic skills and task assessment tool (BRadSTAT), may further enhance evaluation by simultaneously measuring radiologic interpretation and navigational proficiency in reaching peripheral airways [77].

Despite these promising developments, evidence validating AI-guided training in real-world clinical practice remains scarce. Simulation environments cannot yet fully replicate the complexity and unpredictability of patient care, and current findings are largely confined to experimental or educational settings. At present, expert mentorship continues to represent the reference standard for bronchoscopy training and assessment, with AI best regarded as a complementary tool rather than a replacement [78].

## 4. Artificial Intelligence in Endobronchial Ultrasound (EBUS) for Lung Cancer Diagnosis and Staging

EBUS is an advanced bronchoscopic technique that integrates an ultrasound probe, enabling real-time imaging of structures beyond the airway wall. This allows for the visualization of lesions adjacent to the bronchial tree and facilitates transbronchial needle aspiration (EBUS-TBNA) for histopathological sampling [79]. EBUS is particularly valuable for assessing central thoracic lesions, including mediastinal and hilar lymph nodes, making it a critical tool for evaluating nodal metastases and staging lung cancer [80].

Reported diagnostic accuracy of EBUS-TBNA for lung cancer ranges from 81% to 98%, although considerable heterogeneity exists across published studies [81]. In addition to standard grayscale imaging, EBUS can assess vascularity using Doppler ultrasound and tissue stiffness via elastography, further enhancing its diagnostic capabilities [82,83]. For peripheral lesions, a specialized modality known as radial EBUS (rEBUS) enables the localization and sampling of peripheral pulmonary nodules (PPNs), thereby extending the clinical utility of EBUS in lung cancer diagnosis and management [84]. A recent meta-analysis of 41 studies involving 2988 lung nodules reported a pooled diagnostic accuracy of 72.4% (95% CI: 68.7–76.1) for rEBUS, underscoring both its clinical value and its limitations in the evaluation of peripheral lesions [85].

Several sonographic features observed across EBUS modalities are considered indicative of malignant lymph nodes. These include a short-axis diameter greater than 10 mm, absence of a central hilar structure, presence of necrosis, non-hilar vascular patterns, and elastography scores of 4 or 5 [86,87]. Beyond imaging features, blood-based biomarkers and clinical parameters can be integrated to develop predictive models for nodal metastasis [88]. Increasingly, AI models are demonstrating the ability to accurately identify malignant lesions based on ultrasound input alone or by incorporating multimodal diagnostic data [89,90]. Table 3 summarizes key studies that have developed AI models for EBUS image analysis across modalities, including B-mode grayscale, elastography, Doppler ultrasound, and rEBUS, with a specific focus on their application in detecting and diagnosing malignant lymph nodes and PPNs.

Accurate recognition of malignant lesions in EBUS imaging requires reliable detection and segmentation of lymph nodes or lesions. Ervik et al. introduced a U-Net–based framework for automated segmentation of mediastinal lymph nodes and blood vessels in grayscale images, reporting sensitivities of 0.71 ± 0.38 and 0.80 ± 0.25, with specificities of 0.98 ± 0.02 and 0.99 ± 0.01 [91]. Building on this, the group developed a DL model trained on 28,134 EBUS frames to classify lymph node stations, reaching an overall accuracy of 59.5 ± 5.2%, with the best performance at station 4 L and the lowest at station 10 L [92]. These results highlight both the potential and current limitations of automated station recognition, particularly in anatomically complex regions.

In elastography-based EBUS, Zhou et al. proposed a dual-stream feature-fusion attention U-Net (DFA-UNet) integrating convolutional networks with lightweight ViT and hybrid attention mechanisms [93]. Their model outperformed nine state-of-the-art approaches in mediastinal lymph node segmentation, underscoring the value of combining local and global feature extraction to address indistinct boundaries and heterogeneous elasticity. Such developments illustrate rapid progress toward automated multimodal analysis in EBUS, though large-scale validation is still required before routine integration into bronchoscopic workflows.

Early AI applications in EBUS primarily employed ANNs. In 2008, Tagaya et al. trained layered ANNs on B-mode images to distinguish metastatic nodes from sarcoidosis, achieving accuracies of 75.8–91.2% and outperforming thoracic surgeons, whose accuracy was 78% [94]. Ozcelik et al. applied MATLAB-based (version 9.3.0.713579 [R2017b]) ANN modeling to 345 mediastinal nodes, yielding 82% accuracy (sensitivity 89%, specificity 72%, AUC 0.78) [95]. More recently, Koseoglu et al. trained an ML model on 992 nodes and reported 96% accuracy in distinguishing malignant from benign cases [96]. Comparative studies suggest that while ANNs provide strong baseline performance, advanced architectures such as SVMs and DL models can surpass them when trained on curated datasets [96,97].

These findings illustrate the progression from ANN-based classifiers to modern ML frameworks incorporating radiomics and advanced architectures. Although diagnostic accuracies exceeding 90% have been reported, heterogeneity in design, dataset size, and input features limits generalizability. Multi-institutional datasets with rigorous external validation are essential for reproducible performance and clinical translation.

CNN-based models are increasingly applied to grayscale EBUS for metastatic node diagnosis. Churchill et al. evaluated NeuralSeg, training it on 298 nodes and prospectively validating on 108, achieving 72.9% accuracy and 90.8% specificity, thereby demonstrating its utility in ruling out metastasis when biopsy results are inconclusive [98]. Ito et al. trained an Xception-based CNN on more than 5000 frames from 166 nodes, reporting accuracies up to 87.9% and specificities of 95% [99]. Ishiwata et al. used SqueezeNet with transfer learning, achieving 96.7% accuracy with Adam optimization and slightly lower but more stable results with stochastic gradient descent [100]. Yong et al. employed a modified VGG-16 with global average pooling and a custom loss, reaching 75.8% accuracy and an AUC of 0.80, while enabling real-time inference at 63 fps on a single GPU [101].

These approaches reflect a trajectory toward clinical use. Early models were proof-of-concept, while recent work emphasizes robustness through cross-validation, augmentation, and prospective testing. Lightweight architectures such as SqueezeNet demonstrate that high performance can be achieved without excessive computational cost, facilitating real-time deployment on standard GPUs. Despite reported accuracies ranging from 73% to 97%, evidence remains constrained by single-center datasets, modest sample sizes, and limited external validation. Large, multicenter trials with standardized protocols are needed to establish CNNs as reliable adjuncts in nodal staging. Notably, reliance on ultrasound input alone enhances their practicality [102].

Elastography provides a complementary modality by reflecting tissue stiffness, a correlate of malignancy. Zhi et al. developed an ML model for automatic frame selection in strain elastography videos using clustering and color histograms, achieving accuracies of 78–83.5%, comparable to experts and superior to trainees [103]. Xu et al. advanced this with a sparse graph attention mechanism to classify nodes from 727 videos, reaching 81.3% accuracy and an AUC of 0.875 [104]. Patel et al. validated NeuralSeg for lymph node segmentation and stiffness area ratio (SAR) calculation in a prospective trial of 187 nodes, reporting 70.6% accuracy, 90.7% specificity, and an AUC of 0.82, supporting its role in ruling out metastasis when combined with EBUS-TBNA [105].

These studies highlight the potential of AI-enhanced elastography to improve nodal staging through automated frame selection, standardized SAR assessment, and reduced operator dependence. High specificity results warrant further multicenter validation for real-time integration.

Multimodal frameworks further enhance diagnostic performance. Li et al. reported that EBUSNet, combining grayscale, Doppler, and elastography, achieved 88.6% accuracy and an AUC of 0.95, outperforming experts and unimodal models [106]. Lin et al. introduced TransEBUS, a CNN-Transformer hybrid fusing grayscale, Doppler, and elastography with temporal dynamics, achieving 82% accuracy and an AUC of 0.88 [107]. Although multimodal approaches improve predictive reliability, they require longer acquisition and higher computational resources, potentially limiting real-time use.

AI has also been applied to rEBUS for PPN assessment and biopsy guidance. Chen et al. showed that CNNs with transfer learning distinguished benign from malignant lesions with 85.4% accuracy, outperforming texture-based methods [108]. Hotta et al. trained a CNN on over 2.4 million rEBUS frames from 213 patients, reporting 83.4% accuracy and 95.3% sensitivity, surpassing four bronchoscopists whose accuracy was 68.4% [109]. Yu et al. validated a CNN across three centers, reporting an internal AUC of 0.88 and feasibility for malignancy classification and histological subtype prediction, though subtype performance remained modest (AUC 0.64–0.70) [110].

Beyond binary classification, ensemble strategies have shown promise. Khomkham and Lipikorn combined CNNs with radiomic and clinical data in a weighted ensemble with random forests, reaching 95% accuracy and 100% sensitivity in 200 rEBUS images [111]. Xing et al. proposed a fuzzy k-nearest neighbor (FKNN) classifier optimized with a manta ray foraging algorithm, trained on multimodal datasets from 156 patients, achieving 99.4% accuracy in distinguishing malignant from benign lesions [112].

**Table 3:** This table provides an overview of recent research studies that employ AI techniques for the diagnosis and staging of lung cancer, using various forms and modalities of EBUS. A large number of frames derived from only a few patients can artificially inflate reported accuracy, whereas restricting analyses to a single frame per case may reduce the representativeness of the findings. Notably, some models demonstrated higher specificity than sensitivity, while others showed the opposite trend. Diagnostic tools with high sensitivity are valuable for screening, as they minimize the risk of missing affected patients, although confirmatory testing remains necessary. Conversely, tests with high specificity are useful for ruling in disease, meaning that models with very high specificity have the potential to reduce the number of unnecessary biopsies.

Input Data Type	Diagnostic Task	Model Type	Unit of Analysis	Dataset Size	Ground Truth	Performance Metrics	External Validation	Data Availability	Year	Authors [Ref.]
EBUS	Malignant LN recognition	CNN	Patients/frames	773/2569	Histopathology	Acc: 80.6%, Sen: 43.2%, Spe: 96.9%	No	Not available	2024	Patel et al. [102]
EBUS	Malignant LN recognition	CNN	Videos/LNs	53/90	Histopathology	Acc: 96.7%	No	Not available	2024	Ishiwata et al. [100]
EBUS	Malignant LN recognition	SVM	Patients/Lesions	197/205	Histopathology	Acc: 74.2%, Sen: 70.3%, Spe: 74.1%	No	Not available	2024	Hu et al. [97]
EBUS	Malignant LN recognition	SVM/KNN	LNs	992	Histopathology	Acc: 95.9–96.4%	No	Not available	2023	Koseoglu et al. [96]
EBUS	Malignant LN recognition	AE	Patients/LNs	140/298	Histopathology	Acc: 72.9–73.8%	No	Not available	2022	Churchill et al. [98]
EBUS	Malignant LN recognition	CNN	Patients/LNs/frames	91/166/11,699	Histopathology/follow-up	Acc: 87.9%, Sen: 76.9%, Spe: 95.0%	No	Not available	2022	Ito et al. [99]
EBUS	Malignant LN recognition	CNN	LNs/frames	2394/2396	Histopathology	Acc: 75.8%, Sen: 72.7%, Spe: 79.0%	No	On request	2022	Yong et al. [101]
EBUS	Malignant LN recognition	ANN	LNs/frames	345/345	Histopathology/follow-up	Acc: 82%, Sen: 89%, Spe: 72%	No	Not available	2020	Ozcelik et al. [95]
EBUS	Malignant LN recognition	ANN	Patients/LNs	91/91	Histopathology	Acc: 75.8–91.2%, Sen: 84.9–98.5%, Spe: 48–84%	No	Not available	2008	Tagaya et al. [94]
EBUS	LN segmentation	CNN	Patients/frames	56/28,134	Expert consensus	Acc: 59.5%	No	On request	2025	Ervik et al. [92]
EBUS	LN segmentation	AE	Patients/frames	40/1161	Expert consensus	Sen: 71%, Spe: 98%	No	On request	2024	Ervik et al. [91]
EBUS (elastography)	Malignant LN recognition	CNN	Patients/LNs	124/187	Histopathology	Acc: 70.6%, Sen: 43.0%, Spe: 90.7%	No	Not available	2024	Patel et al. [105]
EBUS (elastography)	Malignant LN recognition	CNN	Videos	727	Histopathology/follow-up	Acc: 81.3%	No	Not available	2023	Xu et al. [104]
EBUS (elastography)	Malignant LN recognition	ML	Patients/LNs	351/415	Histopathology/follow-up	Acc: 82.4%	No	On request	2021	Zhi et al. [103]
EBUS (elastography)	LN segmentation	AE, ViT	Patients/frames	206/263	Expert consensus	Prec: 84.4%	No	On request	2024	Zhou et al. [93]
EBUS (gray scale, Doppler, elastography)	Malignant LN recognition	CNN, ViT	Patients/videos	150/330	Histopathology/follow-up	Acc: 82%, Sen: 84.2%, Spe: 80.7%	No	Not available	2025	Lin et al. [107]
EBUS (gray scale, Doppler, elastography)	Malignant LN recognition	AE	Patients/LNs	267/294	Histopathology/follow-up	Acc: 88.6%, Sen: 92.4%, Spe: 83.0%	No	Not available	2021	Li et al. [106]
rEBUS	Malignant PPN recognition	KNN	Patients	156	Histopathology/follow-up	Acc: 99.4%, Sen: 100.0%, Spe: 98.9%	No	Not available	2024	Xing et al. [112]
rEBUS	Malignant PPN recognition	CNN	Patients/PPNs/frames	260/265/769	Histopathology/follow-up	Sen: 58–80%, Spe: 75–92%	Yes	On request	2023	Yu et al. [110]
rEBUS	Malignant PPN recognition	CNN	PPNs	200	Histopathology	Acc: 95%, Sen: 100%, Spe: 86.7%	No	Not available	2022	Khomkham et al. [111]
rEBUS	Malignant PPN recognition	CNN	Patients/frames	213/2421,360	Histopathology/follow-up	Acc: 83.4%, Sen: 95.3%, Spe: 53.6%	No	On request	2022	Hotta et al. [109]
rEBUS	Malignant PPN recognition	CNN	Patients/frames	164/164	Histopathology/follow-up	Acc: 85.4%, Sen: 87.0%, Spe: 82.1%	No	Not available	2019	Chen et al. [108]

Abbreviations: endobronchial ultrasound (EBUS), radial endobronchial ultrasound (rEBUS), lymph nodes (LNs), peripheral pulmonary nodules (PPNs), convolutional neural network (CNN), support vector machine (SVM), autoEncoder (AE), vision transformer (ViT), k-nearest neighbors (KNN), machine learning (ML), artificial neural network (ANN), accuracy (Acc), sensitivity (Sen), specificity (Spe), precision (Prec).

These findings suggest CNN-based methods can surpass physicians in identifying malignant features, while advanced ensembles may further enhance diagnostic power through integration of radiomics and clinical data. Nonetheless, external validation and prospective trials remain essential to ensure robustness and generalizability.

Overall, CNN-based models dominate AI applications in both B-mode EBUS and rEBUS imaging. While elastography and Doppler integration can improve accuracy, their use may prolong procedures. Models combining radiomic and clinical data often yield superior performance, but progress remains limited by scarce public datasets, a lack of open-source code, and insufficient real-world validation. Addressing these challenges will be crucial for establishing AI as a reliable adjunct in EBUS-based nodal staging and lung cancer diagnosis. Similarly to models analyzed in the context of bronchoscopy, studies describing DL systems in EBUS are also subject to potential bias, as large multicenter cohorts remain rare and the literature is dominated by retrospective, single-center designs (Supplementary Figure S2). Prospective multicenter evaluations will be essential to achieve regulatory approval and enable clinical implementation.

## 5. Artificial Intelligence Assistance in Histopathological Examination and Rapid On-Site Evaluation (ROSE)

Although minimally invasive techniques such as bronchoscopy and EBUS offer several advantages over traditional approaches like mediastinoscopy for lung cancer diagnosis, the amount of biopsy material obtained is often limited [113,114]. This limitation poses challenges for pathologists in establishing a definitive diagnosis and may necessitate additional procedures. To address this issue, rapid on-site evaluation (ROSE) was introduced, allowing real-time assessment of sample adequacy during the procedure [115]. Current evidence also suggests that ROSE can reduce the overall cost of EBUS [116]. However, ROSE generally requires the physical presence of a pathologist, which may not always be feasible in clinical practice [117]. Recent studies indicate that AI can effectively analyze ROSE smears, thereby improving both efficiency and diagnostic accuracy in bronchoscopy and EBUS [118,119].

AI algorithms employing CNNs have demonstrated high accuracy in identifying cancerous cells in cytological specimens, with reported performance exceeding 98% [120]. Their efficiency can be further enhanced by integration with automated sample preparation systems, such as ASP Health’s ROSE Prep™ [121]. Notably, diagnostic concordance between AI-driven ROSE systems and experienced cytopathologists in identifying major lung cancer subtypes, including SCC, AC, and SCLC, has shown near perfect agreement [122]. The highest accuracy to date was achieved using cytological images analyzed with a ResNet101-based system, which reported 98.8% accuracy, sensitivity, and specificity [123]. In addition, Wang et al. have proposed the use of whole slide EBUS images to further improve diagnostic performance and processing speed when applying AI models [124].

Despite these encouraging results, expectations should remain cautious. Many reported accuracies approaching 98–99% are derived from retrospective studies of highly curated datasets, which may not capture the variability of routine clinical workflows. Prospective workflow-integrated validations of AI-assisted ROSE remain scarce, and real-world performance across diverse patient populations is yet to be fully established. Consequently, while AI shows considerable potential as an adjunct to ROSE, its role should currently be regarded as supportive rather than definitive until more robust prospective evidence becomes available.

**Figure 4:**
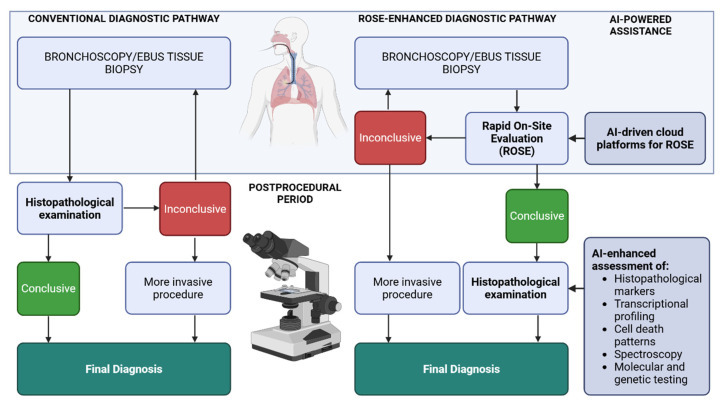
Comparison of the conventional diagnostic pathway relying solely on histopathological examination with the ROSE-enhanced pathway. The steps within the light blue box occur during the endoscopic procedure, whereas those outside the box take place in the postprocedural period. ROSE enables intraoperative tissue assessment, reducing the need for repeated procedures when samples are inconclusive, which is often required in the conventional pathway. The figure highlights key points at which AI-powered solutions can further improve the modern diagnostic workflow following bronchoscopy or EBUS-derived tissue analysis. Created with BioRender.com.

The performance of AI-based ROSE systems may be further optimized by incorporating serum biomarkers and leveraging advanced imaging modalities such as higher harmonic generation microscopy [125,126]. Another promising development is the emergence of cloud-based platforms for AI-powered ROSE assessment, which could improve both accessibility and scalability [127]. A schematic representation of AI applications in ROSE and histopathological analysis within airway endoscopy is provided in Figure 4.

The primary role of ROSE is to confirm sample adequacy and minimize repeat procedures or diagnostic delays. However, definitive diagnosis ultimately depends on comprehensive histopathological assessment, including staining, immunohistochemistry, and molecular or genetic testing [128]. The application of AI in histopathological analysis is expanding across both research and early clinical settings, offering opportunities to support diagnostic accuracy and workflow efficiency [129,130].

Recent advances in ML have contributed to progress in tumor cellularity assessment in clinical practice [131]. Beyond conventional histopathological markers, ML approaches have been investigated for their potential to integrate multidimensional data sources, such as whole-transcriptome RNA sequencing and airway epithelial transcriptional profiling, with the aim of improving risk stratification and diagnostic yield, particularly in patients with indeterminate bronchoscopy results [132,133]. Predictive models based on programmed cell death signatures have also been proposed to generate prognostic indices in lung AC that could eventually inform therapeutic decision-making [134].

Complementary technologies such as RS suggest a role for AI-driven spectral analysis in the rapid detection of malignant lesions, although current evidence remains limited to early studies and controlled research environments [135]. Similarly, ML classifiers trained on histopathological and microenvironmental features from transbronchial lung biopsy specimens have shown promising performance in distinguishing malignant from benign conditions in previously non-diagnostic samples [136].

Taken together, these developments indicate that AI may support the integration of molecular, morphological, and spectroscopic data to advance diagnostic precision and clinical decision-making in thoracic oncology. Nonetheless, most approaches remain at a proof-of-concept stage, and large-scale, workflow-embedded prospective validations are still scarce.

Overall, AI holds promise for augmenting bronchoscopy and EBUS by supporting real-time sample assessment, intraprocedural tissue evaluation, and subsequent histopathological review. These tools could help reduce pathologists’ workload while maintaining diagnostic reliability, though ultimate confirmation continues to rely on comprehensive histopathological evaluation. Beyond sample adequacy assessment, AI may also contribute to the integration of tissue-derived molecular and genomic data. Importantly, rigorous prospective validation remains essential, and the expertise of experienced pathologists continues to be indispensable for ensuring diagnostic accuracy and guiding effective lung cancer management.

## 6. Artificial Intelligence in Other Imaging Modalities and Lung Cancer Screening

The advent of AI has profoundly reshaped medical imaging and its interpretation. While the clinical expertise of radiologists remains indispensable, ML models increasingly complement image analysis across modalities such as CT and positron emission tomography (PET) [137]. These imaging techniques are integral to the diagnosis, staging, and management of lung cancer, providing critical information on tumor localization, morphology, and metabolic activity.

**Figure 5:**
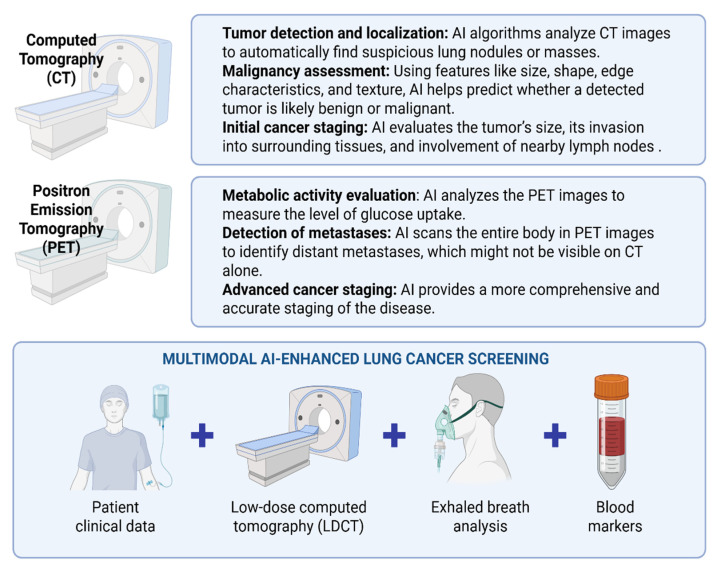
AI integration across various diagnostic tools offers a more comprehensive approach to lung cancer screening. The top panels illustrate AI’s role in analyzing CT and PET images, while the bottom panel highlights a multimodal strategy combining patient clinical data, LDCT, exhaled breath analysis, and blood biomarkers. By merging AI-enhanced insights from both imaging and non-imaging sources, this approach aims to improve early detection, increase diagnostic accuracy, and refine lung cancer staging. Created with BioRender.com.

Although histopathological evaluation of tissue specimens remains the gold standard for definitive diagnosis and treatment planning, imaging features, such as lesion morphology and metabolic behavior, can often suggest malignancy or benignity with considerable reliability [137,138]. In recent years, a growing number of AI-based models have been developed to manage the vast imaging datasets generated in clinical practice. These models are capable of detecting subtle, clinically meaningful patterns that may elude human observers [139,140]. As illustrated in Figure 5, AI-driven approaches can extract diverse diagnostic information from advanced imaging modalities, thereby enhancing lung cancer detection and screening.

For lung cancer staging, PET/CT-based ML classifiers have demonstrated high sensitivity in identifying malignant lymph nodes, frequently achieving low misclassification rates [141,142]. The integration of clinical variables, such as patient demographics and primary tumor characteristics, further improves predictive performance [143,144]. Moreover, certain PET/CT-based AI models can distinguish between histological subtypes of lung cancer [145]. Despite these advances, overall diagnostic accuracy generally remains comparable to that of experienced clinicians. A major barrier to widespread clinical implementation is the lack of large-scale, externally validated studies across diverse patient populations. Emerging evidence also highlights the potential of combining CT-based radiomics with ML algorithms to predict molecular biomarker expression, including PD-L1, EGFR, and Ki-67, as well as functional outcomes such as spirometric indices; however, more research is needed [146,147,148,149,150].

AI applied to low-dose CT (LDCT) is further transforming early lung cancer screening by providing scalable, efficient, and cost-effective solutions [151,152]. Predictive accuracy can be enhanced by integrating additional parameters, including circulating tumor biomarkers and exhaled breath analyses [153,154]. By enabling comprehensive, patient-specific interpretation of large-scale datasets, AI offers the potential for earlier lung cancer detection. This capability may ultimately redefine the paradigm of lung cancer screening and diagnosis, supporting more timely and individualized interventions and improving patient outcomes [155,156].

## 7. Conclusions

AI models applied to bronchoscopy and EBUS have demonstrated considerable potential in lesion recognition, with performance in specific tasks approaching or even exceeding that of experienced clinicians. DL techniques, particularly CNNs, have shown efficacy in processing visual input across multiple modalities, including WLB, AFB, ultrasound, and elastography. Integration with clinical data and radiomics further enhances diagnostic precision, offering the potential to improve biopsy accuracy and, by extension, diagnostic outcomes in lung cancer.

However, despite these advances, important limitations remain. Most models lack external validation, and source code or datasets are rarely made publicly available, restricting reproducibility and the development of more generalizable approaches. The majority of studies are retrospective, single-center, and rely on carefully curated image sets, with poor-quality or complex cases frequently excluded, conditions that do not reflect routine clinical practice. Consequently, while reported performances are often promising, they likely represent optimistic estimates that may not translate directly to real-world clinical settings. Moreover, limited integration with bronchoscopy hardware continues to pose a significant barrier to real-time, intra-procedural application.

Beyond diagnostic assistance, AI holds promise in bronchonavigation and endoscopic education. The concept of navigation based solely on white-light images is particularly intriguing, although current models lack sufficient accuracy for clinical deployment. Established navigation methods, such as fluoroscopy and electromagnetic guidance, remain more reliable. AI is also increasingly used for pre-procedural imaging analysis and intra-procedural histopathological assessment, where it may assist in evaluating sample adequacy and guiding real-time decisions. In the context of education, AI-based tools may support a shift from volume-based to competency-based training by enabling objective, standardized assessment of trainee performance. However, these systems have largely been evaluated in simulated environments, and experienced clinical educators continue to play a critical role in real-world training.

Overall, while AI offers substantial opportunities to enhance bronchoscopy and EBUS across diagnostic, procedural, and educational domains, widespread clinical implementation will depend on rigorous validation, greater data transparency, and closer integration with existing procedural technologies.

## Figures and Tables

**Table 2:** Competency-based training focuses on learners mastering specific skills and progressing by demonstrating their abilities, while volume-based training emphasizes completing a set amount of time or sessions regardless of skill level.

Aspect	Competency-Based Training	Volume-Based Training
*Focus*	Skill mastery and application	Amount of training/time spent
*Progression*	Based on demonstration of skills	Based on time or sessions
*Assessment*	Performance-based	Time or attendance-based
*Pace*	Individualized	Fixed
*Strength*	Ensures readiness and proficiency	Easy to measure and manage

## Data Availability

The datasets analyzed in this study are described in the Supplementary Material. Additional details are available from the corresponding author upon reasonable request.

## Supplementary Materials

The following supporting information can be downloaded at: https://www.mdpi.com/article/10.3390/cancers17172835/s1, Figure S1: Inclusion scheme for relevant publications; Figure S2. Articles from the systematic review were evaluated for bias using QUADAS-2. Most of these studies carry a considerable risk of bias, primarily due to their reliance on small, single-center datasets and retrospective designs. Images are often carefully curated, with poor-quality or complex cases excluded, which does not reflect routine clinical practice. Reference standards are frequently based on expert annotations rather than pathology, while external validation across centers or devices is uncommon, raising concerns about overfitting and limited generalizability. As a result, while reported performances are often promising, they likely represent optimistic estimates that may not translate directly to real-world clinical settings.

## Abbreviations

The following abbreviations are used in this manuscript:

EBUS	endobronchial ultrasound
AI	artificial intelligence
EBUS-TBNA	endobronchial ultrasound-guided transbronchial needle aspiration
ML	machine learning
DL	deep learning
CNN	convolutional neural network
CT	computed tomography
WLB	white light bronchoscopy
AFB	autofluorescence bronchoscopy
NBI	narrow band imaging
RS	Raman spectroscopy
AE	autoEncoder
SVM	support vector machine
Acc	accuracy
AUC	area under the curve
Sen	sensitivity
Spe	specificity
Prec	precision
MARN	multiscale attention residual network
TL	transfer learning
SFT	sequential fine-tuning
PKDN	prior knowledge distillation network
KD MFAD	knowledge distillation-based memory feature unsupervised anomaly detection
DDC	downward deformable convolution
CB Mem	convolutional block focused memory matrix
PPN	peripheral pulmonary nodule
NeRF	neural radiance fields
FBG	fiber Bragg grating
ICU	intensive care unit
BRadSTAT	bronchoscopy–radiologic skills and task assessment tool
rEBUS	radial endobronchial ultrasound
LN	lymph node
ViT	vision transformer
KNN	K-nearest neighbors
ANN	artificial neural network
ROI	region of interest
GPU	graphics processing unit
ROSE	rapid on-site evaluation
PET	positron emission tomography
LDCT	low-dose computed tomography
SCC	squamous cell carcinoma
AC	adenocarcinoma
SCLS	small cell lung carcinoma

## References

[1] Prabhakar B., Shende P., Augustine S. (2018). Current trends and emerging diagnostic techniques for lung cancer. Biomed. Pharmacother..

[2] Dollin Y., Munoz Pineda J.A., Sung L., Hasteh F., Fortich M., Lopez A., Van Nostrand K., Patel N.M., Miller R., Cheng G. (2024). Diagnostic modalities in the mediastinum and the role of bronchoscopy in mediastinal assessment: A narrative review. Mediastinum.

[3] Minami H., Ando Y., Nomura F., Sakai S., Shimokata K. (1994). Interbronchoscopist variability in the diagnosis of lung cancer by flexible bronchoscopy. Chest.

[4] Mwesigwa N.W., Tentzeris V., Gooseman M., Qadri S., Maxine R., Cowen M. (2024). Electromagnetic Navigational Bronchoscopy Learning Curve Regarding Pneumothorax Rate and Diagnostic Yield. Cureus.

[5] Ishiwata T., Yasufuku K. (2024). Artificial intelligence in interventional pulmonology. Curr. Opin. Pulm. Med..

[6] Bertolaccini L., Guarize J., Diotti C., Donghi S.M., Casiraghi M., Mazzella A., Spaggiari L. (2024). Harnessing artificial intelligence for breakthroughs in lung cancer management: Are we ready for the future?. Front. Oncol..

[7] Luo X., Mori K., Peters T.M. (2018). Advanced Endoscopic Navigation: Surgical Big Data, Methodology, and Applications. Annu. Rev. Biomed. Eng..

[8] Shafi S., Parwani A.V. (2023). Artificial intelligence in diagnostic pathology. Diagn. Pathol..

[9] Amisha Malik P., Pathania M., Rathaur V.K. (2019). Overview of artificial intelligence in medicine. J. Fam. Med. Prim. Care.

[10] Bellini V., Cascella M., Cutugno F., Russo M., Lanza R., Compagnone C., Bignami E.G. (2022). Understanding basic principles of Artificial Intelligence: A practical guide for intensivists. Acta Biomed..

[11] Choi R.Y., Coyner A.S., Kalpathy-Cramer J., Chiang M.F., Campbell J.P. (2020). Introduction to Machine Learning, Neural Networks, and Deep Learning. Transl. Vis. Sci. Technol..

[12] Jiang T., Gradus J.L., Rosellini A.J. (2020). Supervised Machine Learning: A Brief Primer. Behav. Ther..

[13] Eckhardt C.M., Madjarova S.J., Williams R.J., Ollivier M., Karlsson J., Pareek A., Nwachukwu B.U. (2023). Unsupervised machine learning methods and emerging applications in healthcare. Knee Surg. Sports Traumatol. Arthrosc..

[14] Al-Hamadani M.N.A., Fadhel M.A., Alzubaidi L., Balazs H. (2024). Reinforcement Learning Algorithms and Applications in Healthcare and Robotics: A Comprehensive and Systematic Review. Sensors.

[15] Sarker I.H. (2021). Deep Learning: A Comprehensive Overview on Techniques, Taxonomy, Applications and Research Directions. SN Comput. Sci..

[16] LeCun Y., Bengio Y., Hinton G. (2015). Deep learning. Nature.

[17] Kufel J., Bargieł-Łączek K., Kocot S., Koźlik M., Bartnikowska W., Janik M., Czogalik Ł., Dudek P., Magiera M., Lis A. (2023). What Is Machine Learning, Artificial Neural Networks and Deep Learning?—Examples of Practical Applications in Medicine. Diagnostics.

[18] Ayachi R., Said Y., Atri M. (2021). A Convolutional Neural Network to Perform Object Detection and Identification in Visual Large-Scale Data. Big Data.

[19] Yamashita R., Nishio M., Do R.K.G., Togashi K. (2018). Convolutional neural networks: An overview and application in radiology. Insights Imaging.

[20] Paul D., Sanap G., Shenoy S., Kalyane D., Kalia K., Tekade R.K. (2021). Artificial intelligence in drug discovery and development. Drug Discov. Today.

[21] Chatterjee S., Das S., Ganguly K., Mandal D. (2024). Advancements in robotic surgery: Innovations, challenges and future prospects. J. Robot. Surg..

[22] Lanotte F., O’Brien M.K., Jayaraman A. (2023). AI in Rehabilitation Medicine: Opportunities and Challenges. Ann. Rehabil. Med..

[23] Olawade D.B., Wada O.J., David-Olawade A.C., Kunonga E., Abaire O., Ling J. (2023). Using artificial intelligence to improve public health: A narrative review. Front. Public. Health.

[24] Bellini V., Valente M., Del Rio P., Bignami E. (2021). Artificial intelligence in thoracic surgery: A narrative review. J. Thorac. Dis..

[25] Mehta V. (2025). Artificial intelligence augmentation raises questions about the future of bronchoscopy. ERJ Open Res..

[26] Lee P., Colt H.G. (2010). Bronchoscopy in lung cancer: Appraisal of current technology and for the future. J. Thorac. Oncol..

[27] Silvestri G.A., Vachani A., Whitney D., Elashoff M., Porta Smith K., Ferguson J.S., Parsons E., Mitra N., Brody J., Lenburg M.E. (2015). A Bronchial Genomic Classifier for the Diagnostic Evaluation of Lung Cancer. N. Engl. J. Med..

[28] Agbontaen K.O., Cold K.M., Woods D., Grover V., Aboumarie H.S., Kaul S., Konge L., Singh S. (2025). Artificial Intelligence-Guided Bronchoscopy is Superior to Human Expert Instruction for the Performance of Critical-Care Physicians: A Randomized Controlled Trial. Crit. Care Med..

[29] Sun W., Yan P., Li M., Li X., Jiang Y., Luo H., Zhao Y. (2024). An accurate prediction for respiratory diseases using deep learning on bronchoscopy diagnosis images. J. Adv. Res..

[30] Deng Y., Chen Y., Xie L., Wang L., Zhan J. (2022). The investigation of construction and clinical application of image recognition technology assisted bronchoscopy diagnostic model of lung cancer. Front. Oncol..

[31] Tan T., Li Z., Liu H., Zanjani F.G., Ouyang Q., Tang Y., Hu Z., Li Q. (2018). Optimize Transfer Learning for Lung Diseases in Bronchoscopy Using a New Concept: Sequential Fine-Tuning. IEEE J. Transl. Eng. Health Med..

[32] Yan P., Sun W., Li X., Li M., Jiang Y., Luo H. (2023). PKDN: Prior Knowledge Distillation Network for bronchoscopy diagnosis. Comput. Biol. Med..

[33] Liu Q., Zheng H., Jia Z., Shi Z. (2025). Tumor detection on bronchoscopic images by unsupervised learning. Sci. Rep..

[34] Vu V.G., Hoang A.D., Phan T.P., Nguyen N.D., Nguyen T.T., Nguyen D.N., Dao N.P., Doan T.P.L., Nguyen T.T.H., Trinh T.H. (2024). BM-BronchoLC—A rich bronchoscopy dataset for anatomical landmarks and lung cancer lesion recognition. Sci. Data.

[35] Cao Y., Zhang J., Zhuo R., Zhao J., Dong Y., Liu T., Zhao H. (2024). BrYOLO-Mamba: A Approach to Efficient Tracheal Lesion Detection in Bronchoscopy. IEEE Access.

[36] Chang Q., Bascom R., Toth J., Ahmad D., Higgins W.E. (2020). Autofluorescence Bronchoscopy Video Analysis for Lesion Frame Detection. Annu. Int. Conf. IEEE Eng. Med. Biol. Soc..

[37] Haritou M., Bountris P., Passalidou E., Koklonis K., Koutsouris D. (2014). An image analysis tool for the classification of lesions suspicious for malignancy in autofluorescence bronchoscopy. Biomed. Spectrosc. Imaging.

[38] Feng P.H., Chen T.T., Lin Y.T., Chiang S.Y., Lo C.M. (2018). Classification of lung cancer subtypes based on autofluorescence bronchoscopic pattern recognition: A preliminary study. Comput. Methods Programs Biomed..

[39] Chang Q., Ahmad D., Toth J., Bascom R., Higgins W.E. (2024). ESFPNet: Efficient Stage-Wise Feature Pyramid on Mix Transformer for Deep Learning-Based Cancer Analysis in Endoscopic Video. J. Imaging.

[40] Daneshpajooh V., Ahmad D., Toth J., Bascom R., Higgins W.E. (2024). Automatic lesion detection for narrow-band imaging bronchoscopy. J. Med. Imaging.

[41] Fousková M., Habartová L., Vališ J., Nahodilová M., Vaňková A., Synytsya A., Šestáková Z., Votruba J., Setnička V. (2024). Raman spectroscopy in lung cancer diagnostics: Can an in vivo setup compete with ex vivo applications?. Spectrochim. Acta A Mol. Biomol. Spectrosc..

[42] Kiraly A.P., Odry B.L., Godoy M.C., Geiger B., Novak C.L., Naidich D.P. (2008). Computer-aided diagnosis of the airways: Beyond nodule detection. J. Thorac. Imaging.

[43] Ramírez E., Sánchez C., Gil D. Localizing Pulmonary Lesions Using Fuzzy Deep Learning. Proceedings of the 2019 21st International Symposium on Symbolic and Numeric Algorithms for Scientific Computing (SYNASC).

[44] Zhang M., Gu Y. (2023). Towards Connectivity-Aware Pulmonary Airway Segmentation. IEEE J. Biomed. Health Inform..

[45] Yang H.W., Wang Y., Zhu H., Zhang J., Deng X., Liu W. A Cascaded Network for Airway Tree Segmentation Incorporating Multiple Attention Mechanisms. Proceedings of the 2024 7th International Symposium on Autonomous Systems (ISAS).

[46] Meng Q., Kitasaka T., Nimura Y., Oda M., Ueno J., Mori K. (2017). Automatic segmentation of airway tree based on local intensity filter and machine learning technique in 3D chest CT volume. Int. J. Comput. Assist. Radiol. Surg..

[47] Mori K., Ota S., Deguchi D., Kitasaka T., Suenaga Y., Iwano S., Hasegawa Y., Takabatake H., Mori M., Natori H. (2009). Automated anatomical labeling of bronchial branches extracted from CT datasets based on machine learning and combination optimization and its application to bronchoscope guidance. Med. Image Comput. Comput. Assist. Interv..

[48] Zhou Z.Q., Guo Z.Y., Zhong C.H., Qiu H.Q., Chen Y., Rao W.Y., Chen X.B., Wu H.K., Tang C.L., Su Z.Q. (2023). Deep Learning-Based Segmentation of Airway Morphology from Endobronchial Optical Coherence Tomography. Respiration.

[49] Li Y., Zheng X., Xie F., Ye L., Bignami E., Tandon Y.K., Rodríguez M., Gu Y., Sun J. (2022). Development and validation of the artificial intelligence (AI)-based diagnostic model for bronchial lumen identification. Transl. Lung Cancer Res..

[50] Chen C., Herth F.J., Zuo Y., Li H., Liang X., Chen Y., Ren J., Jian W., Zhong C., Li S. (2023). Distinguishing bronchoscopically observed anatomical positions of airway under by convolutional neural network. Ther. Adv. Chronic Dis..

[51] Yoo J.Y., Kang S.Y., Park J.S., Cho Y.J., Park S.Y., Yoon H.I., Park S.J., Jeong H.G., Kim T. (2021). Deep learning for anatomical interpretation of video bronchoscopy images. Sci. Rep..

[52] Borrego-Carazo J., Sanchez C., Castells-Rufas D., Carrabina J., Gil D. (2023). BronchoPose: An analysis of data and model configuration for vision-based bronchoscopy pose estimation. Comput. Methods Programs Biomed..

[53] Wang C., Oda M., Hayashi Y., Kitasaka T., Itoh H., Honma H., Takebatake H., Mori M., Natori H., Mori K. (2022). Anatomy Aware-Based 2.5D Bronchoscope Tracking for Image-Guided Bronchoscopic Navigation. Comput. Methods Biomech. Biomed. Eng. Imaging Vis..

[54] Zhu L., Zheng J., Wang C., Jiang J., Song A. (2024). A bronchoscopic navigation method based on neural radiation fields. Int. J. Comput. Assist. Radiol. Surg..

[55] Keuth R., Heinrich M., Eichenlaub M., Himstedt M. (2024). Airway label prediction in video bronchoscopy: Capturing temporal dependencies utilizing anatomical knowledge. Int. J. Comput. Assist. Radiol. Surg..

[56] Cicenia J., Sethi S. (2019). Navigation to peripheral lung nodules using an artificial intelligence-driven augmented image fusion platform (LungVision): A pilot study. Chest.

[57] Whitten P. (2019). Artificial intelligence driven diagnosis of lung cancer in patients with multiple pulmonary nodules. Chest.

[58] Gruionu L.G., Udriștoiu A.L., Iacob A.V., Constantinescu C., Stan R., Gruionu G. (2022). Feasibility of a lung airway navigation system using fiber-Bragg shape sensing and artificial intelligence for early diagnosis of lung cancer. PLoS ONE.

[59] Fried I., Hoelscher J., Akulian J.A., Pizer S., Alterovitz R. Landmark Based Bronchoscope Localization for Needle Insertion Under Respiratory Deformation. Proceedings of the 2023 IEEE/RSJ International Conference on Intelligent Robots and Systems (IROS).

[60] Chahla B., Ozen M. (2024). Fluoroscopy and Cone Beam CT Guidance in Robotic Interventions. Tech. Vasc. Interv. Radiol..

[61] Boac B.M., Kanathanavanich M., Li X., Imai T., Fan X., Walts A.E., Marchevsky A.M., Bose S. (2024). Accuracy and efficacy of Ion robotic-assisted bronchoscopic fine needle aspiration of lung lesions. J. Am. Soc. Cytopathol..

[62] Zhang J., Liu L., Xiang P., Fang Q., Nie X., Ma H., Hu J., Xiong R., Wang Y., Lu H. (2024). AI co-pilot bronchoscope robot. Nat. Commun..

[63] Mitros Z., Thamo B., Bergeles C., da Cruz L., Dhaliwal K., Khadem M. (2021). Design and Modelling of a Continuum Robot for Distal Lung Sampling in Mechanically Ventilated Patients in Critical Care. Front. Robot. AI.

[64] Xu S., Wang X., Qin Y., Wang H., Yu N., Han J. Depth-Awareness Shared Self-Supervised Bronchial Orifice Segmentation for Center Detection in Vision-Based Robotic Bronchoscopy. Proceedings of the 2024 IEEE 14th International Conference on CYBER Technology in Automation, Control, and Intelligent Systems (CYBER).

[65] Zheng M., Ye M., Rafii-Tari H. Automatic Biopsy Tool Presence and Episode Recognition in Robotic Bronchoscopy Using a Multi-Task Vision Transformer Network. Proceedings of the 2022 International Conference on Robotics and Automation (ICRA).

[66] Zhao J., Chen H., Tian Q., Chen J., Yang B., Zhang Z., Liu H. BronchoCopilot: Towards Autonomous Robotic Bronchoscopy via Multimodal Reinforcement Learning. Proceedings of the 2024 IEEE/RSJ International Conference on Intelligent Robots and Systems (IROS).

[67] Sachdeva A., Sethi S. (2024). Motivation and Learning: Leveraging Artificial Intelligence to Improve Bronchoscopy Performance. Chest.

[68] Hostetter L.J., Nelson D.R. (2025). Competency-based medical education in interventional pulmonology: Current state and future opportunities. Curr. Opin. Pulm. Med..

[69] Healy W.J., Musani A., Fallaw D.J., Islam S.U. (2024). Emerging Role of Artificial Intelligence in Academic Pulmonary Medicine. South. Med. J..

[70] Cold K.M., Vamadevan A., Nielsen A.O., Konge L., Clementsen P.F. (2023). Systematic Bronchoscopy: The Four Landmarks Approach. J. Vis. Exp..

[71] Cold K.M., Agbontaen K., Nielsen A.O., Andersen C.S., Singh S., Konge L. (2025). Artificial intelligence improves bronchoscopy performance: A randomised crossover trial. ERJ Open Res..

[72] Cold K.M., Xie S., Nielsen A.O., Clementsen P.F., Konge L. (2024). Artificial Intelligence Improves Novices’ Bronchoscopy Performance: A Randomized Controlled Trial in a Simulated Setting. Chest.

[73] Cold K.M., Wei W., Agbontaen K., Singh S., Konge L. (2025). Mastery Learning Guided by Artificial Intelligence Is Superior to Directed Self-Regulated Learning in Flexible Bronchoscopy Training: An RCT. Respiration.

[74] Xu W., Hou G., Deng M. (2024). A novel artificial intelligence feedback system boosts novice bronchoscopy performance based on chest-CT simulated tracheal models: A randomized controlled trial. Eur. Respir. J..

[75] Mora A., Debiasi E. (2023). Leveraging artificial intelligence for development of a cost-effective bronchoscopy simulator for resource-constrained settings. Chest.

[76] Cold K.M., Agbontaen K., Nielsen A.O., Andersen C.S., Singh S., Konge L. (2024). Artificial intelligence for automatic and objective assessment of competencies in flexible bronchoscopy. J. Thorac. Dis..

[77] Yap E.L.C., Vandal A.C., Williamson J.P., Nguyen P., Colt H. (2022). Development of a Bronchoscopy-Radiologic Skills and Task Assessment Tool (BRadSTAT): A Tool for Evaluating the Radiological Skills of Bronchoscopists with Different Experience. Respiration.

[78] Huang J., Lin J., Lin Z., Li S., Zhong C. (2024). Artificial Intelligence Feedback for Bronchoscopy Training: Old Wine in a New Bottle or True Innovation?. Chest.

[79] Jaliawala H.A., Farooqui S.M., Harris K., Abdo T., Keddissi J.I., Youness H.A. (2021). Endobronchial Ultrasound-Guided Transbronchial Needle Aspiration (EBUS-TBNA): Technical Updates and Pathological Yield. Diagnostics.

[80] Fielding D.I., Kurimoto N. (2013). EBUS-TBNA/staging of lung cancer. Clin. Chest Med..

[81] Torre M., Reda M., Musso V., Danuzzo F., Mohamed S., Conforti S. (2021). Diagnostic accuracy of endobronchial ultrasound-transbronchial needle aspiration (EBUS-TBNA) for mediastinal lymph node staging of lung cancer. Mediastinum.

[82] Zhou X., Li Y. (2025). Diagnostic utility of endobronchial ultrasound elastography for detecting benign and malignant lymph nodes: A retrospective study. J. Thorac. Dis..

[83] Nosotti M., Palleschi A., Tosi D., Mendogni P., Righi I., Carrinola R., Rosso L. (2017). Color-Doppler sonography patterns in endobronchial ultrasound-guided transbronchial needle aspiration of mediastinal lymph-nodes. J. Thorac. Dis..

[84] Chen A., Chenna P., Loiselle A., Massoni J., Mayse M., Misselhorn D. (2014). Radial probe endobronchial ultrasound for peripheral pulmonary lesions. A 5-year institutional experience. Ann. Am. Thorac. Soc..

[85] McGuire A.L., Myers R., Grant K., Lam S., Yee J. (2020). The Diagnostic Accuracy and Sensitivity for Malignancy of Radial-Endobronchial Ultrasound and Electromagnetic Navigation Bronchoscopy for Sampling of Peripheral Pulmonary Lesions: Systematic Review and Meta-analysis. J. Bronchol. Interv. Pulmonol..

[86] Wang B., Guo Q., Wang J.Y., Yu Y., Yi A.J., Cui X.W., Dietrich C.F. (2021). Ultrasound Elastography for the Evaluation of Lymph Nodes. Front. Oncol..

[87] Huang Z., Wang L., Chen J., Zhi X., Sun J. (2024). A risk-scoring model based on endobronchial ultrasound multimodal imaging for predicting metastatic lymph nodes in lung cancer patients. Endosc. Ultrasound.

[88] Choi J., Zo S., Kim J.H., Oh Y.J., Ahn J.H., Kim M., Lee K., Lee H.Y. (2023). Nondiagnostic, radial-probe endobronchial ultrasound-guided biopsy for peripheral lung lesions: The added value of radiomics from ultrasound imaging for predicting malignancy. Thorac. Cancer.

[89] Wu J., Wu C., Zhou C., Zheng W., Li P. (2021). Recent advances in convex probe endobronchial ultrasound: A narrative review. Ann. Transl. Med..

[90] Achim C., Rusu-Both R., Chira R.I. Computerised application for lung cancer diagnosis based on transthoracic ultrasonography. Proceedings of the 2018 IEEE-EMBS Conference on Biomedical Engineering and Sciences (IECBES).

[91] Ervik Ø., Tveten I., Hofstad E.F., Langø T., Leira H.O., Amundsen T., Sorger H. (2024). Automatic Segmentation of Mediastinal Lymph Nodes and Blood Vessels in Endobronchial Ultrasound (EBUS) Images Using Deep Learning. J. Imaging.

[92] Ervik Ø., Rødde M., Hofstad E.F., Tveten I., Langø T., Leira H.O., Amundsen T., Sorger H. (2025). A New Deep Learning-Based Method for Automated Identification of Thoracic Lymph Node Stations in Endobronchial Ultrasound (EBUS): A Proof-of-Concept Study. J. Imaging.

[93] Zhou Q., Zhou Y., Hou N., Zhang Y., Zhu G., Li L. (2024). DFA-UNet: Dual-stream feature-fusion attention U-Net for lymph node segmentation in lung cancer diagnosis. Front. Neurosci..

[94] Tagaya R., Kurimoto N., Osada H., Kobayashi A. (2008). Automatic objective diagnosis of lymph nodal disease by B-mode images from convex-type echobronchoscopy. Chest.

[95] Ozcelik N., Ozcelik A.E., Bulbul Y., Oztuna F., Ozlu T. (2020). Can artificial intelligence distinguish between malignant and benign mediastinal lymph nodes using sonographic features on EBUS images?. Curr. Med. Res. Opin..

[96] Koseoglu F.D., Alıcı I.O., Er O. (2023). Machine learning approaches in the interpretation of endobronchial ultrasound images: A comparative analysis. Surg. Endosc..

[97] Hu W., Wen F., Zhao M., Li X., Luo P., Jiang G., Yang H., Herth F.J.F., Zhang X., Zhang Q. (2024). Endobronchial Ultrasound-Based Support Vector Machine Model for Differentiating between Benign and Malignant Mediastinal and Hilar Lymph Nodes. Respiration.

[98] Churchill I.F., Gatti A.A., Hylton D.A., Sullivan K.A., Patel Y.S., Leontiadis G.I., Farrokhyar F., Hanna W.C. (2022). An Artificial Intelligence Algorithm to Predict Nodal Metastasis in Lung Cancer. Ann. Thorac. Surg..

[99] Ito Y., Nakajima T., Inage T., Otsuka T., Sata Y., Tanaka K., Sakairi Y., Suzuki H., Yoshino I. (2022). Prediction of Nodal Metastasis in Lung Cancer Using Deep Learning of Endobronchial Ultrasound Images. Cancers.

[100] Ishiwata T., Inage T., Aragaki M., Gregor A., Chen Z., Bernards N., Kafi K., Yasufuku K. (2024). Deep learning-based prediction of nodal metastasis in lung cancer using endobronchial ultrasound. JTCVS Tech..

[101] Yong S.H., Lee S.H., Oh S.I., Keum J.S., Kim K.N., Park M.S., Chang Y.S., Kim E.Y. (2022). Malignant thoracic lymph node classification with deep convolutional neural networks on real-time endobronchial ultrasound (EBUS) images. Transl. Lung Cancer Res..

[102] Patel Y.S., Gatti A.A., Farrokhyar F., Xie F., Hanna W.C. (2024). Artificial Intelligence Algorithm Can Predict Lymph Node Malignancy from Endobronchial Ultrasound Transbronchial Needle Aspiration Images for Non-Small Cell Lung Cancer. Respiration.

[103] Zhi X., Li J., Chen J., Wang L., Xie F., Dai W., Sun J., Xiong H. (2021). Automatic Image Selection Model Based on Machine Learning for Endobronchial Ultrasound Strain Elastography Videos. Front. Oncol..

[104] Xu M., Chen J., Li J., Zhi X., Dai W., Sun J., Xiong H. (2023). Automatic Representative Frame Selection and Intrathoracic Lymph Node Diagnosis With Endobronchial Ultrasound Elastography Videos. IEEE J. Biomed. Health Inform..

[105] Patel Y.S., Gatti A.A., Farrokhyar F., Xie F., Hanna W.C. (2024). Clinical utility of artificial intelligence-augmented endobronchial ultrasound elastography in lymph node staging for lung cancer. JTCVS Tech..

[106] Li J., Zhi X., Chen J., Wang L., Xu M., Dai W., Sun J., Xiong H. (2021). Deep learning with convex probe endobronchial ultrasound multimodal imaging: A validated tool for automated intrathoracic lymph nodes diagnosis. Endosc. Ultrasound.

[107] Lin C.K., Wu S.H., Chua Y.W., Fan H.J., Cheng Y.C. (2025). TransEBUS: The interpretation of endobronchial ultrasound image using hybrid transformer for differentiating malignant and benign mediastinal lesions. J. Formos. Med. Assoc..

[108] Chen C.H., Lee Y.W., Huang Y.S., Lan W.R., Chang R.F., Tu C.Y., Chen C.Y., Liao W.C. (2019). Computer-aided diagnosis of endobronchial ultrasound images using convolutional neural network. Comput. Methods Programs Biomed..

[109] Hotta T., Kurimoto N., Shiratsuki Y., Amano Y., Hamaguchi M., Tanino A., Tsubata Y., Isobe T. (2022). Deep learning-based diagnosis from endobronchial ultrasonography images of pulmonary lesions. Sci. Rep..

[110] Yu K.L., Tseng Y.S., Yang H.C., Liu C.J., Kuo P.C., Lee M.R., Huang C.T., Kuo L.C., Wang J.Y., Ho C.C. (2023). Deep learning with test-time augmentation for radial endobronchial ultrasound image differentiation: A multicentre verification study. BMJ Open Respir. Res..

[111] Khomkham B., Lipikorn R. (2022). Pulmonary Lesion Classification Framework Using the Weighted Ensemble Classification with Random Forest and CNN Models for EBUS Images. Diagnostics.

[112] Xing J., Li C., Wu P., Cai X., Ouyang J. (2024). Optimized fuzzy K-nearest neighbor approach for accurate lung cancer prediction based on radial endobronchial ultrasonography. Comput. Biol. Med..

[113] Um S.W., Kim H.K., Jung S.H., Han J., Lee K.J., Park H.Y., Choi Y.S., Shim Y.M., Ahn M.J., Park K. (2015). Endobronchial ultrasound versus mediastinoscopy for mediastinal nodal staging of non-small-cell lung cancer. J. Thorac. Oncol..

[114] Czarnecka-Kujawa K., Yasufuku K. (2017). The role of endobronchial ultrasound versus mediastinoscopy for non-small cell lung cancer. J. Thorac. Dis..

[115] Jain D., Allen T.C., Aisner D.L., Beasley M.B., Cagle P.T., Capelozzi V.L., Hariri L.P., Lantuejoul S., Miller R., Mino-Kenudson M. (2018). Rapid On-Site Evaluation of Endobronchial Ultrasound-Guided Transbronchial Needle Aspirations for the Diagnosis of Lung Cancer: A Perspective From Members of the Pulmonary Pathology Society. Arch. Pathol. Lab. Med..

[116] Kalluri M., Puttagunta L., Ohinmaa A., Thanh N.X., Wong E. (2015). Cost Analysis of Intra Procedural Rapid on Site Evaluation of Cytopathology with Endobronchial Ultrasound. Int. J. Technol. Assess. Health Care.

[117] Witt B.L. (2021). Rapid On Site Evaluation (ROSE): A Pathologists’ Perspective. Tech. Vasc. Interv. Radiol..

[118] Asfahan S., Elhence P., Dutt N., Niwas Jalandra R., Chauhan N.K. (2021). Digital-Rapid On-site Examination in Endobronchial Ultrasound-Guided Transbronchial Needle Aspiration (DEBUT): A proof of concept study for the application of artificial intelligence in the bronchoscopy suite. Eur. Respir. J..

[119] Koratala A., Chandra N.C., Pulipaka S.P., Colleti S., Lee-Mateus A.Y., Barrios-Ruiz A., Neshat S., Diaz-Churion F., Johnson M.M., Abia Trujillo D. (2023). Artificial Intelligence in Rapid On-Site Evaluation of Bronchoscopy Samples. Am. J. Respir. Crit. Care Med..

[120] Lan H., Chen P., Wang C., Chen C., Yao C., Jin F., Wan T., Lv X., Wang J. (2024). A Multiscale Connected UNet for the Segmentation of Lung Cancer Cells in Pathology Sections Stained Using Rapid On-Site Cytopathological Evaluation. Am. J. Pathol..

[121] Subramanian H., Oleari N., Bluestone A., Danczuk J., Randolph M., Costaldi M. (2024). Improving the Efficiency of Rapid Onsite Evaluation Utilizing Artificial Intelligence. J. Am. Soc. Cytopathol..

[122] Yan S., Li Y., Pan L., Jiang H., Gong L., Jin F. (2024). The application of artificial intelligence for Rapid On-Site Evaluation during flexible bronchoscopy. Front. Oncol..

[123] Lin C.K., Chang J., Huang C.C., Wen Y.F., Ho C.C., Cheng Y.C. (2021). Effectiveness of convolutional neural networks in the interpretation of pulmonary cytologic images in endobronchial ultrasound procedures. Cancer Med..

[124] Wang C.W., Khalil M.A., Lin Y.J., Lee Y.C., Huang T.W., Chao T.K. (2022). Deep Learning Using Endobronchial-Ultrasound-Guided Transbronchial Needle Aspiration Image to Improve the Overall Diagnostic Yield of Sampling Mediastinal Lymphadenopathy. Diagnostics.

[125] Chen J., Zhang C., Xie J., Zheng X., Gu P., Liu S., Zhou Y., Wu J., Chen Y., Wang Y. (2024). Automatic lung cancer subtyping using rapid on-site evaluation slides and serum biological markers. Respir. Res..

[126] van Huizen L.M.G., Blokker M., Daniels J.M.A., Radonic T., von der Thüsen J.H., Veta M., Annema J.T., Groot M.L. (2025). Rapid On-Site Histology of Lung and Pleural Biopsies Using Higher Harmonic Generation Microscopy and Artificial Intelligence Analysis. Mod. Pathol..

[127] Zhang S., Raff R., Rossi J., Zukovsky E., Thosani N. (2023). Rapid Onsite Evaluation (ROSE) Anywhere and Anytime: Developing a Cloud Based Artificial Intelligence (AI) Platform Service. J. Am. Soc. Cytopathol..

[128] Travis W.D. (2020). Lung Cancer Pathology: Current Concepts. Clin. Chest Med..

[129] Hays P. (2024). Artificial intelligence in cytopathological applications for cancer: A review of accuracy and analytic validity. Eur. J. Med. Res..

[130] Vaickus L.J., Kerr D.A., Velez Torres J.M., Levy J. (2024). Artificial Intelligence Applications in Cytopathology: Current State of the Art. Surg. Pathol. Clin..

[131] Kiyuna T., Cosatto E., Hatanaka K.C., Yokose T., Tsuta K., Motoi N., Makita K., Shimizu A., Shinohara T., Suzuki A. (2024). Evaluating Cellularity Estimation Methods: Comparing AI Counting with Pathologists’ Visual Estimates. Diagnostics.

[132] Tomlinson G.S., Thomas N., Chain B.M., Best K., Simpson N., Hardavella G., Brown J., Bhowmik A., Navani N., Janes S.M. (2016). Transcriptional Profiling of Endobronchial Ultrasound-Guided Lymph Node Samples Aids Diagnosis of Mediastinal Lymphadenopathy. Chest.

[133] Choi Y., Qu J., Wu S., Hao Y., Zhang J., Ning J., Yang X., Lofaro L., Pankratz D.G., Babiarz J. (2020). Improving lung cancer risk stratification leveraging whole transcriptome RNA sequencing and machine learning across multiple cohorts. BMC Med. Genomics..

[134] Wang S., Wang R., Hu D., Zhang C., Cao P., Huang J. (2024). Machine learning reveals diverse cell death patterns in lung adenocarcinoma prognosis and therapy. NPJ Precis. Oncol..

[135] Leblond F., Dallaire F., Tran T., Yadav R., Aubertin K., Goudie E., Romeo P., Kent C., Leduc C., Liberman M. (2023). Subsecond lung cancer detection within a heterogeneous background of normal and benign tissue using single-point Raman spectroscopy. J. Biomed. Opt..

[136] Sano H., Okoshi E.N., Tachibana Y., Tanaka T., Lami K., Uegami W., Ohta Y., Brcic L., Bychkov A., Fukuoka J. (2024). Machine-Learning-Based Classification Model to Address Diagnostic Challenges in Transbronchial Lung Biopsy. Cancers.

[137] Guglielmo P., Marturano F., Bettinelli A., Sepulcri M., Pasello G., Gregianin M., Paiusco M., Evangelista L. (2023). Additional Value of PET and CT Image-Based Features in the Detection of Occult Lymph Node Metastases in Lung Cancer: A Systematic Review of the Literature. Diagnostics.

[138] Flechsig P., Frank P., Kratochwil C., Antoch G., Rath D., Moltz J., Rieser M., Warth A., Kauczor H.U., Schwartz L.H. (2017). Radiomic Analysis using Density Threshold for FDG-PET/CT-Based N-Staging in Lung Cancer Patients. Mol. Imaging Biol..

[139] Kawaguchi Y., Matsuura Y., Kondo Y., Ichinose J., Nakao M., Okumura S., Mun M. (2021). The predictive power of artificial intelligence on mediastinal lymphnode metastasis. Gen. Thorac. Cardiovasc. Surg..

[140] Teramoto A., Tsujimoto M., Inoue T., Tsukamoto T., Imaizumi K., Toyama H., Saito K., Fujita H. (2019). Automated Classification of Pulmonary Nodules through a Retrospective Analysis of Conventional CT and Two-phase PET Images in Patients Undergoing Biopsy. Asia Ocean. J. Nucl. Med. Biol..

[141] Guberina M., Herrmann K., Pöttgen C., Guberina N., Hautzel H., Gauler T., Ploenes T., Umutlu L., Wetter A., Theegarten D. (2022). Prediction of malignant lymph nodes in NSCLC by machine-learning classifiers using EBUS-TBNA and PET/CT. Sci. Rep..

[142] Rogasch J.M.M., Michaels L., Baumgärtner G.L., Frost N., Rückert J.C., Neudecker J., Ochsenreither S., Gerhold M., Schmidt B., Schneider P. (2023). A machine learning tool to improve prediction of mediastinal lymph node metastases in non-small cell lung cancer using routinely obtainable [18F]FDG-PET/CT parameters. Eur. J. Nucl. Med. Mol. Imaging.

[143] Laros S.S.A., Dieckens D., Blazis S.P., van der Heide J.A. (2022). Machine learning classification of mediastinal lymph node metastasis in NSCLC: A multicentre study in a Western European patient population. EJNMMI Phys..

[144] Ouyang M.L., Wang Y.R., Deng Q.S., Zhu Y.F., Zhao Z.H., Wang L., Wang L.X., Tang K. (2021). Development and Validation of a 18F-FDG PET-Based Radiomic Model for Evaluating Hypermetabolic Mediastinal-Hilar Lymph Nodes in Non-Small-Cell Lung Cancer. Front. Oncol..

[145] Zhou Y., Ma X.L., Zhang T., Wang J., Zhang T., Tian R. (2021). Use of radiomics based on 18F-FDG PET/CT and machine learning methods to aid clinical decision-making in the classification of solitary pulmonary lesions: An innovative approach. Eur. J. Nucl. Med. Mol. Imaging.

[146] Hashimoto K., Murakami Y., Omura K., Takahashi H., Suzuki R., Yoshioka Y., Oguchi M., Ichinose J., Matsuura Y., Nakao M. (2024). Prediction of Tumor PD-L1 Expression in Resectable Non-Small Cell Lung Cancer by Machine Learning Models Based on Clinical and Radiological Features: Performance Comparison With Preoperative Biopsy. Clin. Lung Cancer.

[147] Digumarthy S.R., Padole A.M., Gullo R.L., Sequist L.V., Kalra M.K. (2019). Can CT radiomic analysis in NSCLC predict histology and EGFR mutation status?. Medicine.

[148] Yamazaki M., Yagi T., Tominaga M., Minato K., Ishikawa H. (2022). Role of intratumoral and peritumoral CT radiomics for the prediction of EGFR gene mutation in primary lung cancer. Br. J. Radiol..

[149] Sun H., Zhou P., Chen G., Dai Z., Song P., Yao J. (2023). Radiomics nomogram for the prediction of Ki-67 index in advanced non-small cell lung cancer based on dual-phase enhanced computed tomography. J. Cancer Res. Clin. Oncol..

[150] Boulogne L.H., Charbonnier J.P., Jacobs C., van der Heijden E.H.F.M., van Ginneken B. (2024). Estimating lung function from computed tomography at the patient and lobe level using machine learning. Med. Phys..

[151] Ziegelmayer S., Graf M., Makowski M., Gawlitza J., Gassert F. (2022). Cost-Effectiveness of Artificial Intelligence Support in Computed Tomography-Based Lung Cancer Screening. Cancers.

[152] Trujillo J.C., Soriano J.B., Marzo M., Higuera O., Gorospe L., Pajares V., Olmedo M.E., Arrabal N., Flores A., García J.F. (2025). Cost-effectiveness of a machine learning risk prediction model (LungFlag) in the selection of high-risk individuals for non-small cell lung cancer screening in Spain. J. Med. Econ..

[153] Ye M., Tong L., Zheng X., Wang H., Zhou H., Zhu X., Zhou C., Zhao P., Wang Y., Wang Q. (2022). A Classifier for Improving Early Lung Cancer Diagnosis Incorporating Artificial Intelligence and Liquid Biopsy. Front. Oncol..

[154] Mohamed E.I., Mohamed M.A., Abdel-Mageed S.M., Abdel-Mohdy T.S., Badawi M.I., Darwish S.H. (2019). Volatile organic compounds of biofluids for detecting lung cancer by an electronic nose based on artificial neural network. J. Appl. Biomed..

[155] Hesso I., Kayyali R., Zacharias L., Charalambous A., Lavdaniti M., Stalika E., Ajami T., Acampa W., Boban J., Gebara S.N. (2024). Cancer care pathways across seven countries in Europe: What are the current obstacles? And how can artificial intelligence help?. J. Cancer Policy.

[156] Purohit L., Kiamos A., Ali S., Alvarez-Pinzon A.M., Raez L. (2025). Incidental Pulmonary Nodule (IPN) Programs Working Together with Lung Cancer Screening and Artificial Intelligence to Increase Lung Cancer Detection. Cancers.

