# Supplementary material for: Integrating Artificial Intelligence in Bronchoscopy and Endobronchial Ultrasound (EBUS) for Lung Cancer Diagnosis and Staging: A Comprehensive Review
Source: Cancers (Basel). 2025 Aug 29;17(17):2835. doi: 10.3390/cancers17172835 (PMC12427279; doi:10.3390/cancers17172835)
Supplement: Supplementary file 1 [file cancers-17-02835-s001.zip › cancers-3841929-supplementary.pdf]

cancers

MDPI

Supplementary Materials

Integrating Artificial Intelligence in Bronchoscopy and Endobronchial Ultrasound (EBUS) for Lung Cancer Diagnosis and Staging: A Comprehensive Review

Supplementary Materials and Methods:

Literature search

The selected literature (n = 2,116) was retrieved from PubMed, Embase, and IEEE Xplore (accessed on 16 March 2025) without any time frame restrictions. Search terms, combined in various configurations using the Boolean operators “AND” and “OR,” were as follows:

"Artificial Intelligence", "AI", "Machine Learning", "Deep Learning", "Neural Networks", "Deep Neural Networks", "Convolutional Neural Networks", "CNN", "Radiomics", "Computer-Aided Diagnosis", "CAD", "Automated Detection", "Automated Image Analysis", "Natural Language Processing", "NLP", "Big Data", "Predictive Modeling", "Decision Support Systems", "Medical Image Processing", "Bronchoscopy", "Bronchoscopic", "Endobronchial Ultrasound", "EBUS", "Linear EBUS", "Radial EBUS", "Airway Navigation", "Virtual Bronchoscopy", "Robotic Bronchoscopy", "Robotic-Assisted Bronchoscopy", "Transbronchial Biopsy", "TBNA", "Transbronchial Needle Aspiration", "Mediastinal Staging", "Lung Nodule Biopsy", "Interventional Pulmonology", "Navigational Bronchoscopy", "Electromagnetic Navigation Bronchoscopy", "ENB", "Fluorescence Bronchoscopy", "Augmented Reality Bronchoscopy".

Literature screening

After the final selection, duplicate studies and articles published in languages other than English were removed. Articles with abstracts unrelated to any type of artificial intelligence (AI) were excluded, along with research papers discussing AI in contexts not involving any modality of bronchoscopy or endobronchial ultrasound (EBUS). One article could not be retrieved, leaving 129 papers for eligibility assessment. Articles unrelated to lung cancer were subsequently excluded. In addition, reviews, commentaries, and editorials were omitted, resulting in 35 articles selected for final analysis. The literature review was conducted from April to July 2025, and the screening process is illustrated in **Figure S1**.

Both abstracts and full texts were independently screened by two teams, each comprising one clinician and one AI professional. The inter-rater agreement between the two teams, measured using Cohen’s κ, was 0.82, indicating substantial agreement. Any disagreements were resolved by a senior authors’ committee, which included both an experienced clinician and an AI expert.

A total of 35 articles identified through systematic literature screening were assessed for risk of bias using the QUADAS-2 tool, with the results presented in **Figure S2**.

To enhance readability and comprehensively cover the topic of artificial intelligence (AI) in airway endoscopy, the article was structured as a narrative review. The systematic review formed the core of the analysis, while manual screening was employed to broaden the scope and include closely related topics.

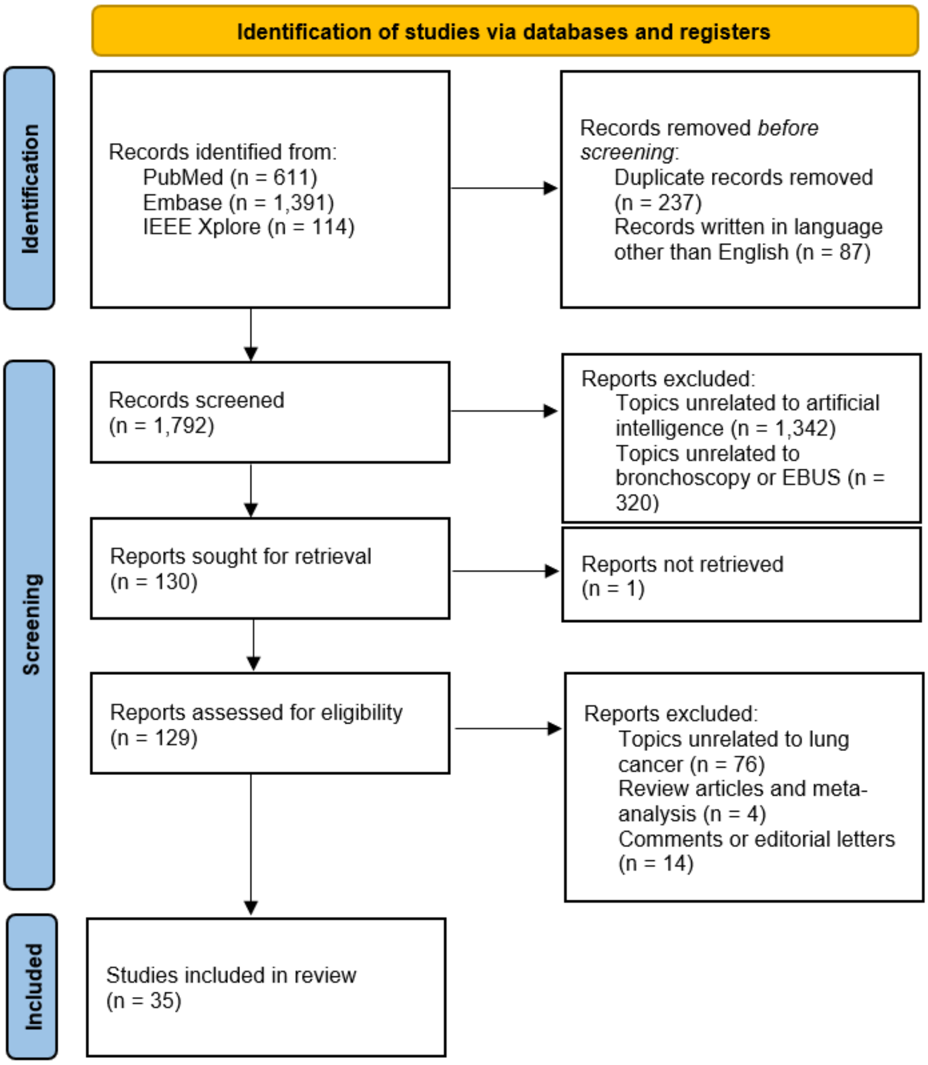

Figure S1. Inclusion scheme for relevant publications.

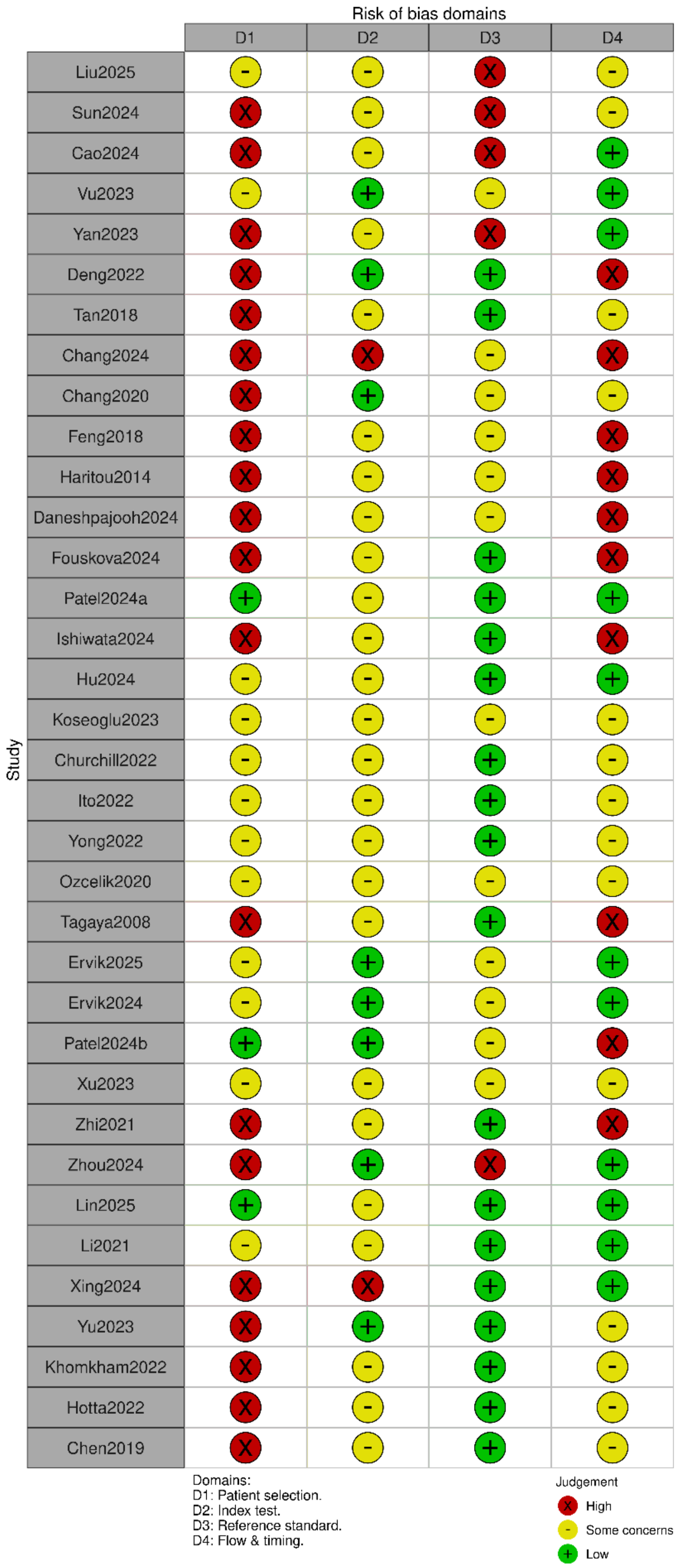

Figure S2. Articles from the systematic review were evaluated for bias using QUADAS-2. Most of these studies carry a considerable risk of bias, primarily due to their reliance on small, single-center datasets and retrospective designs. Images are often carefully curated, with poor-quality or complex

cases excluded, which does not reflect routine clinical practice. Reference standards are frequently based on expert annotations rather than pathology, while external validation across centers or devices is uncommon, raising concerns about overfitting and limited generalizability. As a result, while reported performances are often promising, they likely represent optimistic estimates that may not translate directly to real-world clinical settings.

 https://doi.org/10.3390/xxxxx
